# Comprehensive Genetic Analysis of DGAT2 Mutations and Gene Expression Patterns in Human Cancers

**DOI:** 10.3390/biology10080714

**Published:** 2021-07-26

**Authors:** Meghan Graber, Hayley Barta, Ryan Wood, Amrit Pappula, Martin Vo, Ruben C. Petreaca, Wilber Escorcia

**Affiliations:** 1 Biology Department, Xavier University, Cincinnati, OH 45207, USA; graberm2@xavier.edu (M.G.); bartah@xavier.edu (H.B.); woodr9@xavier.edu (R.W.); vom@xavier.edu (M.V.); 2 Computer Science and Engineering Undergraduate Program, The Ohio State University, Columbus, OH 43210, USA; pappula.1@buckeyemail.osu.edu; 3 Department of Molecular Genetics, The Ohio State University, Marion, OH 43302, USA

**Keywords:** lipid metabolism, lipid droplet, lipid storage, DNA damage, mutation, gene expression, cancer

## Abstract

**Simple Summary:**

Cellular metabolism including lipid metabolism is often altered in cancer cells. To keep up with the high energetic demand of cancer cells lipid metabolism is increased. Thus, mutations, changes in gene expression or other alterations of lipid metabolism genes are often seen in cancer cells. In this report we characterized DGAT2, a gene required for triacylglycerol synthesis and cell membrane structure using the Catalogue of Somatic Mutations in Cancers (COSMIC). We identified a hotspot mutation at D222V that may affect enzyme activity in cancer cells. Additionally, we find that DGAT2 mutations in cancer cells are distinguishable from a conserved mutation that is linked to Axonal Charcot-Marie-Tooth disease, an inherited condition leading to muscle degeneration. This suggests that DGAT2 mutations and alterations in cancer cells are specific to drive cellular transformation and immortalization.

**Abstract:**

DGAT2 is a transmembrane protein encoded by the DGAT2 gene that functions in lipid metabolism, triacylglycerol synthesis, and lipid droplet regulation. Cancer cells exhibit altered lipid metabolism and mutations in DGAT2 may contribute to this state. Using data from the Catalogue of Somatic Mutations in Cancer (COSMIC), we analyzed all cancer genetic DGAT2 alterations, including mutations, copy number variations and gene expression. We find that several DGAT2 mutations fall within the catalytic site of the enzyme. Using the Variant Effect Scoring Tool (VEST), we identify multiple mutations with a high likelihood of contributing to cellular transformation. We also found that D222V is a mutation hotspot neighboring a previously discovered Y223H mutation that causes Axonal Charcot-Marie-Tooth disease. Remarkably, Y223H has not been detected in cancers, suggesting that it is inhibitory to cancer progression. We also identify several single nucleotide polymorphisms (SNP) with high VEST scores, indicating that certain alleles in human populations have a pathogenic predisposition. Most mutations do not correlate with a change in gene expression, nor is gene expression dependent on high allele copy number. However, we did identify eight alleles with high expression levels, suggesting that at least in certain cases, the excess DGAT2 gene product is not inhibitory to cellular proliferation. This work uncovers unknown functions of DGAT2 in cancers and suggests that its role may be more complex than previously appreciated.

## 1. Introduction

Lipids play many important roles in the cell, including energy storage, structure, signaling, gene regulation, and metabolism [1]. Although much is known about the reprogramming of lipid metabolism by cancer cells, such as upregulated fatty acid oxidation and cholesterol accumulation [2], the roles of many lipid regulators involved in specific genomic instability processes have only recently been extensively characterized [3,4,5]. One major category of lipids is nonmembrane lipids, which are primarily neutral lipids such as fatty acids (FAs) and derivatives such as triacylglycerol. Triacylglycerols are often found sequestered into lipid droplets (LDs) and form the pool of free FAs for mitochondrial beta oxidation [6].

Triacylglycerol biosynthesis follows either the glycerol phosphate pathway or the monoacylglycerol pathway [7]. Within both pathways, the acyl CoA diacylglycerol acyltransferase enzymes DGAT1 and DGAT2 catalyze the conversion of diacylglycerol to triacylglycerol. Both enzymes are found on the surface of the ER membrane, with DGAT1 on the luminal side and DGAT2 facing the cytoplasmic side [8].

While DGAT1 has additional functions, DGAT2 is specific to triacylglycerol synthesis. It catalyzes a covalent bond between de novo synthesized activated fatty acids (FA-CoA) and diacylglycerol [9]. DGAT2 is of crucial importance to cell membrane stability. Knocking out DGAT2 in mice is lethal because it leads to impaired skin permeability and dehydration [8]. DGAT2 mutations are also associated with physiological alterations like Charcot-Marie-Tooth disease in humans, compromising muscle structure and function [10].

Lipid droplets are primarily composed of triacylglycerols and other neutral lipids [11]. These lipids are enclosed by a phospholipid monolayer, creating a droplet that can store nonpolar molecules in the aqueous cytosol [12]. Lipid droplets have a variety of functions, including preventing cellular damage by sequestering lipotoxic molecules, providing FAs for energy production, and maintaining ER and membrane homeostasis [13].

Lipid droplet metabolism involves the balance between lipolysis, lipophagy, and lipogenesis. Lipolysis releases FAs from lipid droplets through a series of sequential enzymes that break down triacylglycerols [13]. In adipose tissues, the products are released into the bloodstream, while in nonadipose tissues, the FAs are used for beta oxidation [14].

Lipid droplets are also degraded in autophagic compartments by lytic enzymes [14]. An overabundance of free FAs causes an increase in lipid droplet synthesis, which protects the cell from damaging lipotoxic effects [13]. This protective role and the ability of LDs to efficiently transfer FAs to the mitochondria ensure that LD biogenesis occurs even during periods of nutrient deprivation, using autophagy of structural lipids [13].

Cancer cells exhibit the Warburg effect in which glycolytic products are diverted away from the Krebs Cycle [15]. Instead of undergoing normal cellular respiration when oxygen is present, cancer metabolism prioritizes fermentation. This increases glutamine metabolism, lactate production, and gluconeogenesis, a process that results in elevated lipogenic precursors such as citrate. Consequently, cancer cells show greater lipid synthesis to meet the increased energetic demands of tumors [16]. This metabolic shift is characterized by *de novo* synthesis of FAs, which can be converted to triacylglycerol by DGAT2 and stored in lipid droplets. Many cancers have been found to display accumulation of lipid droplets, including those affecting brain, lung, renal, and skin tissues [13].

DGAT2 mutations in cancers are common but have not yet been thoroughly investigated. In this study, we carried out a pan-cancer analysis of DGAT2 mutations. Using data from the Catalogue of Somatic Mutations in Cancers (COSMIC) [17], we analyzed the mutational landscape of DGAT2 and characterized pathogenic mutations associated with changes in DGAT2 function that likely contribute to cellular transformation.

## 2. Materials and Methods

COSMIC is a repository for somatic mutations and genome alterations in cancers. It combines data from the NIH Cancer Genome Atlas (TCGA) project, the International Cancer Genome Consortium (ICGC), the Cell Lines Project, as well as other independent studies [17]. The COSMIC data is manually curated by experts and often includes PubMed IDs of studies which we used for references in this report. An Excel file with all DGAT2 COSMIC mutations was downloaded from https://cancer.sanger.ac.uk/cosmic (version 92, accessed on 29 October 2020) and statistically analyzed to reveal mutation frequencies that may affect the functional role of DGAT2 in cancer metabolism. The copy number variation file was generated using the Copy Number Analysis (CONAN) tool. Expression data of DGAT2 was also downloaded from the same site.

Protein alignments of the DGAT2 isoforms were generated using COBALT (https://www.ncbi.nlm.nih.gov/tools/cobalt/re_cobalt.cgi, accessed on 4 March 2021) as described previously [18,19]. The lollipop figures were made as reported earlier [20,21].

The Cancer-Related Analysis of Variants Toolkit (CRAVAT) software was employed to determine the likelihood of mutations being drivers or passengers and was used as previously described [22,23].

All statistical analyses and graphs were done using SPSS^®^ version 17. The figures were created in Photoshop^®^.

## 3. Results and Discussion

### 3.1. DGAT2 Isoforms and Conservation

The DGAT2 gene contains eight introns and nine exons [24]. Two accepted transcript variants and protein isoforms and several alternatively spliced isoforms have been reported (Table 1). The major transcript reported on COSMIC (accessed in October 2020) is NM_032564.5 (ENST00000228027.11), corresponding to isoform 1. However, an isoform 2, as well as a predicted transcript X1, are also listed on NCBI. All amino acid annotations/mutations listed in this report use isoform 1. An alignment of the major DGAT2 isoforms shows that a central region is conserved among all of them (Supplementary Figure S1). Previous studies have also shown that DGAT2 is ubiquitous in both plants and animals [24,25]. A comprehensive study of 117 sequences from 70 organisms showed that DGAT2 is more conserved than DGAT1 [26]. An alignment of several animal sequences shows lower conservation in the first 40 amino acids (Supplementary Figure S1, Table S1). Additionally, human Isoform X1 appears to be the major transcript in several other species suggesting that the N-terminus of the protein is more plastic and often functionally dispensable.

### 3.2. DGAT2 Mutation Distribution in Human Cancers

**Figure 1:**
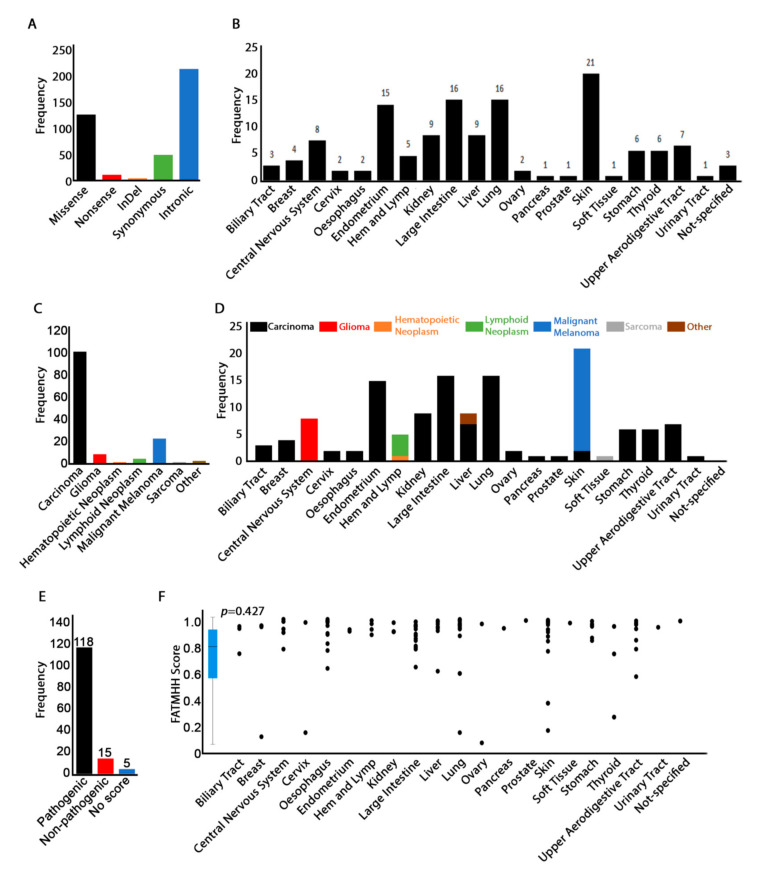
Distribution of DGAT2 mutations in cancer samples. (**A**) Frequency of mutation types in all cancer samples reported on COSMIC. “Intronic” mutations also include 5′ and 3′ UTRs. (**B**) Tissue distribution of missense, non-sense and indel mutations. Synonymous, intronic and UTR mutations were excluded from this analysis as they are not likely to change the function of the gene. The panel follows the same color coding as in (**A**). (**C**) Distribution of missense, non-sense and InDel mutations by cancer histology. (**D**) Distribution of cancer histology by tissue. This panel follows the same color coding as in (**C**). (**E**) Frequency of missense, non-sense and InDel pathogenic mutations. (**F**) Scatter graph to show tissue correlation with the FATMHH score. The distribution of the FATHMM score was also graphed as a boxplot for easy visualization of median and quartiles. Probability values represent Pearson’s two-tailed significance.

The COSMIC database reports a total of 398 DGAT2 mutations detected in 21 different cancers (Figure 1A,B and Supplementary Table S2). The majority of the mutations within DGAT2 are intronic, substitution/missense, and synonymous (Figure 1A). Non-sense mutations also represent frameshifts resulting from insertions and deletions introducing a stop codon before the endogenous stop codon. The highest frequency of missense mutations was observed in skin tissue samples (frequency (f) = 21). Other tissues showing elevated mutation frequencies include the lungs (f = 16), large intestines (f = 16), and endometrium (f = 15) (Figure 1B). The cancer type for some mutations is not specified. Consistent with the high mutation frequencies seen in epithelial tissues, the most common cancer type associated with DGAT2 mutations was carcinoma, followed by malignant melanoma (Figure 1C). This result also correlates with a high prevalence of cancer in skin, lung, large intestine, and endometrial tissues (Figure 1D).

### 3.3. Mutation Pathogenicity Is Correlated with Older Age

COSMIC reports a FATHMM score that indicates the pathogenicity of a mutation [27]. Scores range between 0 and 1 and mutations with a score of 0.7 and higher are predicted to be pathogenic. A graph of all FATMHH scores of DGAT2 CDS mutations (excluding intronic and 5′ and 3′ UTRs) shows that most of the missense, non-sense, and InDel mutations were associated with high pathogenicity scores (Figure 1E). Pathogenic mutations were identified in all cancers, but esophagus, large intestine, lung, and skin tissue mutations were characterized by clusters of high pathogenic scores (Figure 1F). This heavy burden of pathogenic mutations within the coding sequence is expected considering the high level of DGAT2 sequence conservation. Consistent with the general lack of sequence conservation in the N-terminus, the average FATMHH score for mutations within the first 39 amino acids is low, but variability is high (0.48 ± 0.37). This suggests that mutations in certain key residues are highly pathogenic while others are not. Predictably, the pathogenic score for all other residues is substantial (0.77 ± 0.3).

Genomic instability is a defining feature of cancer cells and a driving force behind tumor evolution [28]. It is also an established hallmark of aging [29]. The accumulation of genetic damage both results from and contributes to the aging process [30]. COSMIC reports patient age at the time of tissue extraction and sequencing for most samples. Thus, we investigated any correlation between pathogenic mutations and patient age.

**Figure 2:**
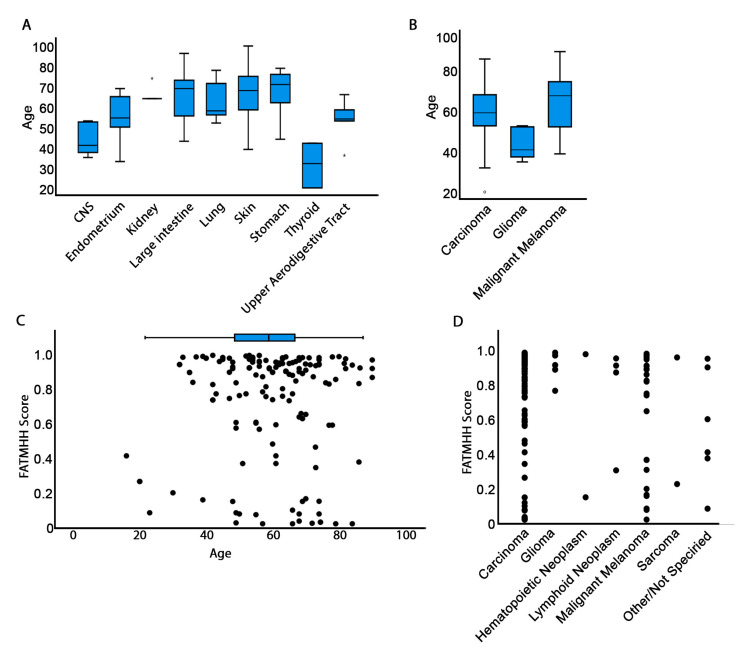
Correlation of patient age with mutation pathogenicity. (**A**) Age and tissue distribution of mutations. (**B**) Age and histology distribution of mutations. Only tissues for which at least five samples were reported were included in (**A**) and (**B**). Complete data are reported in Figure S2. For A and B, circles represent outliners and asterisks extreme values. (**C**) Scatter graph to show the correlation of FATMHH pathogenicity score and age. (**D**) Scatter graph to show the distribution of FATMHH pathogenicity score by cancer histology.

DGAT2 mutations linked to elevated pathogenicity were observed in samples from patients whose ages were near the post-reproductive period or in the senescent stage of life (Figure 2 and Supplementary Figure S2). For instance, endometrium samples were taken from patients in the 40–50 age range (Figure 2A). Patients with reported ages of 50–55 years contributed samples with prevalent DGAT2 mutations in large intestine and skin tissues, while stomach mutations originated primarily in samples from septuagenarians. Thyroid cancers mostly revealed DGAT2 mutations in specimens from younger individuals. Similarly, in gliomas, DGAT2 mutations were mainly detected in subjects around the age of 40. The significance of these observations is not immediately clear due to the limitations of small sample sizes for these tissues.

Carcinomas and malignant melanomas were linked to DGAT2 mutations in samples from patients in the 50–70 age group (Figure 2B). These results agree with the expected increase of cancer prevalence observed during senescence. Intriguingly, the young age of patients whose samples were linked to gliomas (~40 years) may indicate a set point in mid-maturity when alterations to DGAT2 can result from and contribute to oncogenic states in the brain. However, these age data are interpreted with some caution because it is not clear from COSMIC whether the age reported in the database occurs at diagnosis or following patient death. Moreover, COSMIC data does not reveal age of cancer onset, which limits potential inferences to relative comparisons with published data on the subject.

The high pathogenicity scores of DGAT2 mutations in carcinoma samples (above 0.9625) and in many tissue types, notably skin and esophagus, suggests that the DGAT2 mutations in these tissues may be critical for cellular immortalization or cancer progression. Shifts in lipid metabolism are an established hallmark of cancer [2]. Thus, given the crucial involvement of DGAT2 in lipid regulation, pathogenic mutations in this gene may contribute to shifts in lipid metabolism that are implicated in promotion of cancer. Our findings on DGAT2 mutation pathogenicity in carcinomas support previous research on hepatocellular carcinomas (HCC). DGAT2 was found to be downregulated in HCC, while increased DGAT2 expression repressed tumor growth [31]. The DGAT2 mutational landscape includes alterations that may decrease or inhibit its enzymatic activity, resulting in hypomorphic DGAT2 in the cell. This would create a similar effect to that of DGAT2 downregulation observed in hepatocellular carcinomas and suggests an important role of DGAT2 functional insufficiency in cancer progression.

To further understand the contribution of DGAT2 mutations to carcinogenesis, we used the Cancer-Related Analysis of Variants Toolkit (CRAVAT) software which predicts the potential of a mutation to be driver or pathogenic [22,32,33,34]. All COSMIC-reported mutations (including noncoding/intronic) were processed using this analysis. Because of the small data set, we extracted only mutations with p-values lower than 0.05 and false discovery rates (FDR) lower than 0.01 (Supplementary Table S3).

Our analysis reveals that none of the DGAT2 mutations (coding or noncoding) are drivers. This was an expected outcome because, to our knowledge, this gene has not been characterized as a driver gene. However, certain mutations including D222V (see Section 3.3) were pathogenic within statistical significance (Table 2). Notably, all pathogenic mutations fall within the DGAT2 catalytic site, and they do not correlate with any tissue specificity. None of the intronic or 5′ and 3′ UTR mutations are pathogenic. We also observed that high pathogenicity correlates well with specimens from patients older than 40 years of age (Figure 2C). In general, however, carcinomas and melanomas, which are exceedingly represented in samples from older patients, are statistically enriched in higher levels of pathogenic mutations (Figure 2D).

Multiple DGAT2 mutations have previously been identified as single nucleotide polymorphisms (SNPs) by COSMIC as well as NCBI (Supplementary Table S3), including eight that the CRAVAT algorithm identifies as pathogenic (Table 2). NCBI does not list clinical significance for any of the eight SNPs nor could we find any literature describing the phenotypes of these alleles. However, one publication does report several intergenic SNPs associated with decreased DGAT2 expression, which promotes predisposition to prostate cancer [35]. We investigated expression data for DGAT2 mutations (see Section 3.7) and indeed, one of the alleles (R218W, rs528376420) appears to show increased expression levels, though the results are highly variable and not statistically significant under our analysis.

Taken together, these data show that certain DGAT2 mutations are likely to promote cellular transformation. Moreover, certain SNPs in human populations may predispose individuals to cancer development. However, the clinical significance of these SNPs remains to be further investigated.

### 3.4. Identification of a Mutation Hotspot in DGAT2

**Figure 3:**
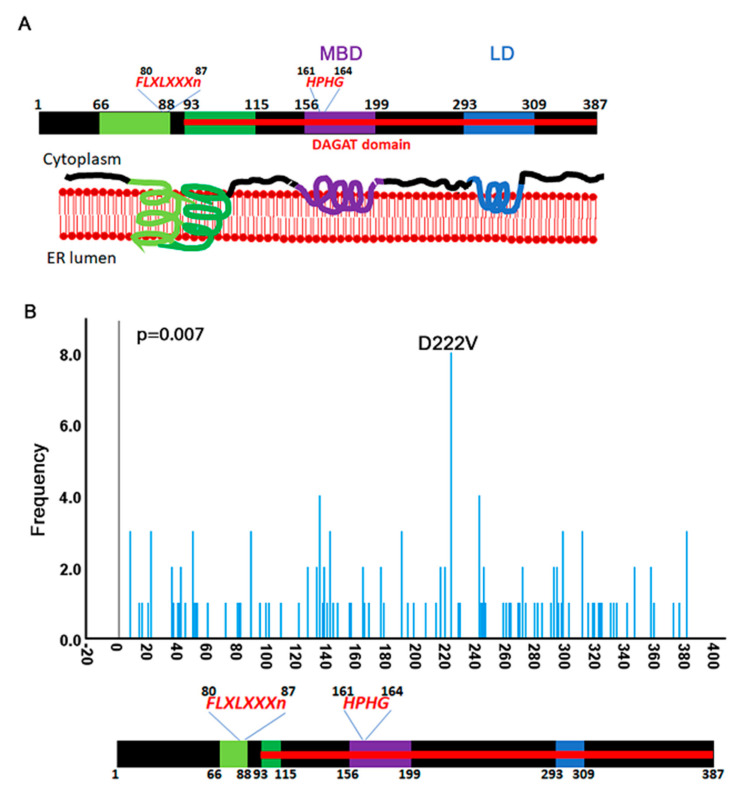
Mutation distribution over the open reading frame of DGAT2. (**A**) Cartoon of DGAT2 protein structure adapted from [7]. Residue numbers are inferred from the mouse data. The protein is predicted to cross the ER membrane once between amino acids 66–115 and bind (but not cross) the membrane two more times between 156–119 and 293–309. The amino acid sequence HPHG is conserved in the DGAT2 family proteins. The FLXLXXXn is a consensus sequence for lipid metabolizing proteins. (B) Frequency distribution of mutations that fall over the CDS of DGAT2. A D222B hotspot is identified in kidney cancers.

DGAT2 is a transmembrane protein with two domains embedded in the phospholipid bilayer on the N-terminal half [7,36] (Figure 3A). Two other domains towards the C-terminus are also embedded but do not cross the entire membrane. The C-terminus of DGAT2 is necessary for the catalytic function of the enzyme [37]. Several conserved domains have been identified within the C-terminus: the membrane-binding domain (MBD) from residues 156 to 199 interacts with the ER membrane, and the lipid droplet (LD) targeting domain from 293–309, interacts with lipid droplets. These findings are consistent with a recent report that identified three cysteine residues in the C-terminus at 172, 214, and 312 that are essential for DGAT2 catalytic activity [9]. The catalytic domain of the enzyme spans between amino acids 93 and 387. Two conserved consensus sequences HPHG (conserved in DGAT family proteins) [36] and FLXLXXXn (conserved in lipid metabolizing enzymes) [38] have also been identified. This structure of the protein is inferred from mouse data and simulated models [7,36] because, to our knowledge, it has not yet been determined in humans.

A visual inspection of COSMIC mutations shows that they are distributed throughout the entire region of the coding sequence. However, the Kolmogorov–Smirnov test for uniformity reveals a statistically nonuniform distribution (*p* = 0.007). Indeed, we identified a D222V hotspot which appears primarily in renal carcinomas (Figure 3B) [39]. Remarkably, a recent report characterized a mutation in the neighboring residue (Y223H) in a family with Axonal Charcot-Marie-Tooth disease [10]. The authors propose that this mutation explains the autosomal dominant inheritance of the disease. Phenotypes associated with p.Y223H in CMT include decreased serum TG levels and decreased axonal branching. Both the D222 and Y223 residues are highly conserved in animals (Supplementary Figure S1).

However, a mutation at Y223 does not appear in cancers reported on COSMIC. Overexpression of the DGAT2 Y223H mutant inhibited cellular proliferation [10], so it is possible that it negatively affects cancer progression and is therefore selected against. An analysis of mutation preferences in cancer cells shows that Y to H (tyrosine to histidine) mutations are twice as likely to appear in cancers than D to V (aspartate to valine) [40], which would suggest that the Y223H mutation should be more likely than D222V. Y to H mutations also have a higher evolution tendency than D to V [41]. Thus, this strongly indicates that mutation of the Y223 residue affects the function of the protein in a manner that is inhibitory to cancer development.

### 3.5. Mutational Landscape

**Figure 4:**
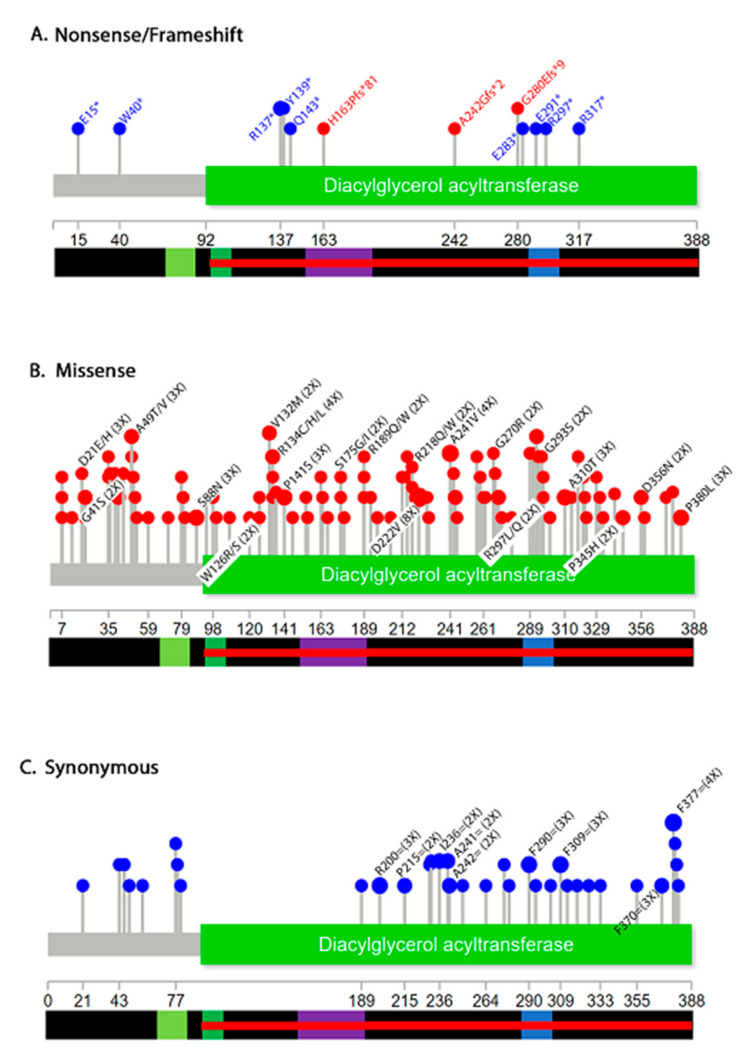
Distribution of DGAT mutations by mutation type. Shown are non-sense (blue) and frameshift (red) (**A**), missense (**B**) and synonymous (**C**). Only mutations that appear more than twice are labeled. Complete data is shown in Supplementary Table S2.

We next investigated the distribution of DGAT2 mutations by mutation type (Figure 4). Several non-sense mutations, including three frameshifts that introduce a stop codon were identified (Figure 4A). These mutations appear in multiple cancer types: hematopoietic and lymphoid (A242Gfs*2), urinary tract (E15*), lung (W40*, E283*, E291*), endometrium (H163Pfs*81), skin (Q143*, R137*), stomach (R297*), large intestine (R317*) and esophagus (Y139*). For one, the tissue is not specified (G280EFs*9). Mutations that appear early in the sequence are predicted to have a more drastic effect because they truncate larger regions of the protein. Non-sense mutations (p.E15*, p.W40*, p.R137*, p.Y139*, and p.Q143*) may truncate the protein before the MBD and LD targeting domains. Additionally, mutations (p.E283*, p.E291*, and p.R297*) may result in an incomplete LD targeting domain. A frameshift insertion (H163Pfs*81) within the MBD segment, an insertion before the LD domain (A242Gfs*2), and a deletion (G280Efs*9) could also impact the normal function of DGAT2. Unfortunately, COSMIC does not provide information on zygosity of these mutations, so it is unclear if one or both alleles of the gene are affected. Therefore, in this analysis, we cannot rule out changes to DGAT2 function that stem from haploinsufficiency.

Missense mutations may also impact the catalytic activity in the C-terminus. As previously mentioned, the p.Y223H mutation contributes to the human disease Charcot-Marie-Tooth disease (CMT) [10]. Of the cancer samples found in COSMIC, the highest frequency missense mutation was p.D222V, occurring just before the defining CMT residue and within the same well-conserved N-acetyltransferase superfamily (NAT-SF) domain (Figure 4B). Several other mutations with a frequency ≥2 appear within the catalytic subunit of the enzyme and within or near the MBD or LD domains (Figure 4B). Some substitutions such as W126R/S, G270R, G293S, A310T, P345H and P380L cause a change from polar to nonpolar residues or vice versa. This observation indicates potential changes in structure that could impact the enzymatic function of the protein.

Synonymous mutations do not alter protein sequence but may affect mRNA stability or codon usage during translation [42]. Thus, they can affect gene expression. A graph of synonymous mutations shows a complete absence in the middle of the protein between amino acids 77–189 (Figure 4C). The reason for this is unclear but suggests that nucleotide alterations may not be tolerated in that region.

### 3.6. DGAT2 Expression Levels in Cancer Tissues

Gene expression levels are available for certain TCGA samples. We retrieved DGAT2 expression levels for the various cohorts listed on COSMIC (Supplementary Figure S3). The data is represented as a Z-score, which is internally normalized to control [43]. If there is no change in gene expression compared to control the Z-value is 0. Z-values greater than +2 or lower than −2 represent over-expression or under-expression, respectively. Our analysis of the various cohorts shows that all are skewed towards over-expressed DGAT2, suggesting that certain individuals within the groups have high expression levels of the gene. This does not appear to be cancer specific. We found this paradoxical since several studies have shown that DGAT2 overexpression inhibits cell proliferation [10,31,35].

To understand whether DGAT2 mutations affect gene expression levels, we extracted Z-values for all TCGA samples (Supplementary Table S4). We find that most mutation values are within the normal range. We identified 10 mutations that were over-expressed (Table 3).

Because Z-values are internally controlled, expression data of individual samples may only be compared within the cohort. When compared with the cohort range, eight out of the 10 mutations showed increased expression levels (Table 3). We searched the literature to see if expression levels for the 10 mutations were previously characterized. TCGA P141S expression levels were determined in lung squamous cell carcinoma. Additionally, a study on BRAF^V600^ melanoma patients who received vemurafenib and dabrafenib chemotherapy also identified the mutation [44]. However, the melanoma study did not determine expression levels. Four different mutations at the R189 residues have been identified: R189=, R189L and R189Q [45] in the skin; and R189W in the endometrium. Only the TCGA data lists expression levels for W40*, G167D, I94V and for the two noncoding mutations *27A>G and *556A>T. These analyses indicate that most DGAT2 mutations do not affect gene expression.

### 3.7. Copy Number Variations of DGAT2 Alleles

**Figure 5:**
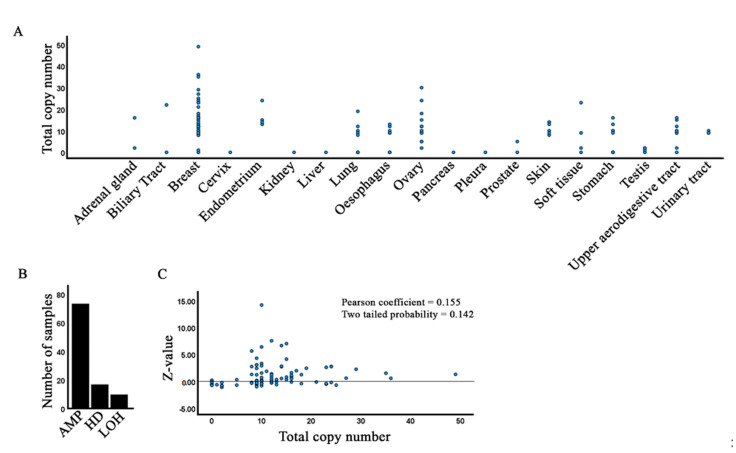
Copy number variation of DGAT2 alleles. (**A**) Copy number extracted from COSMIC with the CONAN tool. (**B**) Distribution of DGAT2 amplification (AMP), homozygous deletion (HD) and loss of heterozygosity (LOH) in CONAN samples. (**C**) Correlation of copy number with expression level (Z-value).

We next used the Copy Number Analysis (CONAN) tool to investigate whether cancers are characterized by variations in DGAT2 copy numbers. Indeed, we find that most cancer tissues feature an increased copy number of DGAT2, including some samples with substantial increases (e.g., breast and ovary) (Figure 5A). NCBI reports the highest DGAT2 expression levels in fat tissue followed by liver and skin. The other tissues are characterized by low gene expression [46]. A higher copy number may allow higher gene expression in certain cancers. Nonetheless, an increased copy number does not appear to correlate with either the type of cancer or with tissues where normal expression is high.

To investigate if copy number translates to higher expression, we extracted Z-values for samples derived from CONAN (Supplementary Table S5). Our analysis shows that there is no correlation between copy number and gene expression (Figure 5C). Remarkably, gene expression is highest when copy number is 10–20. Further experimental work is necessary to reveal the functional implications of this observation. A smaller subset of samples is characterized by homozygous deletion, which suggests that in those contexts, DGAT2 is not essential for cell proliferation or cancer progression.

## 4. Conclusions

**Figure 6:**
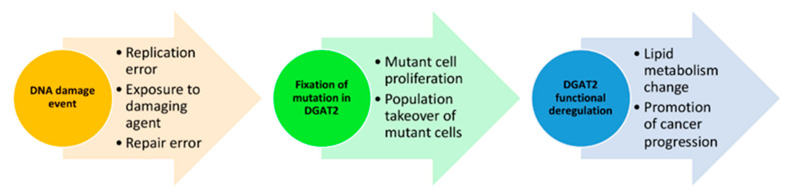
Model of the functional consequence in cancers linked to DGAT2 mutations. An initial DNA damage event on the sequence of DGAT2 occurs through various mechanisms. DGAT2 pathogenic mutations become fixed in cells that adapt and thrive in specific tissue environments. As the population of DGAT2 mutant cells outcompetes its wild-type counterparts, lipid metabolism changes due to altered function in the DGAT2 protein and associated lipid regulators. Cancer-promoting conditions select for DGAT2 deregulation to contribute to cellular transformation.

Analyzing the mutational landscape of more regulators such as DGAT2 increases our understanding of deregulated lipid metabolism in cancer. Our analysis identified several things about mutated DGAT2 in cancer tissues. First, mutations distribute evenly throughout the CDS sequence. This is an expected observation given the high conservation of DGAT2 sequence and function. Second, DGAT2 mutations are found in all cancer types suggesting that deregulated gene activity is not cancer specific. Third, we identify pathogenic mutations and the likelihood that certain SNPs may predispose individuals to cancer. Fourth, expression levels are not associated with cancer type or allele copy number. Taken together, these data suggest that the role of deregulated DGAT2 is context-dependent across cancers and is associated with structural alterations in conserved functional domains (Figure 6). Future examination of mutations in other lipid regulators will help determine the impact of deregulated lipid metabolism on cancer progression and facilitate a probabilistic assessment of cancer predisposition following post-biopsy sequencing.

## Figures and Tables

**Table 1:** DGAT2 Transcripts and isoforms.

^1^ Transcript	Transcript Length (Nucleotides)	^2^ Protein Isoforms	Protein Size (Amino Acids)	UniProt Identifier
NM_032564.5	2407	1	388	Q96PD7-1
NM_001253891.2	2278	2	345	Q96PD7-2
XM_011545304	2156	X1	358	N/A
-	-	3	295	S4R3S3
-	-	4	160	S4R383
-	-	5	112	S4R3Z3
-	-	6	113	S4R449

^1^ The variants are from NCBI. ^2^ The isoforms 1 and 2 are accepted isoforms from NCBI. Isoform X1 is predicted. Isoforms 3–6 are possible isoforms from UniProt.

**Table 2:** DGAT2 alleles with significant VEST values.

^1^ Mutation	^2^ VEST *p*-Value	^2^ VEST FDR	Tissue	^3^ dbSNP
G212C	0.00071000	0.05	CNS	rs777087960
H163R	0.00071000	0.05	Endometrium	
R259H	0.00091000	0.05	Large Intestine	
E243Q	0.00121000	0.05	Upper Aer Dig	
F314S	0.00152000	0.05	Upper Aer Dig	
P329S	0.00182000	0.05	Endometrium	
F262V	0.00182000	0.05	Pancreas	
G261C	0.00253000	0.05	Lung	
G197D	0.00283000	0.05	Endometrium	rs369680804
S278F	0.00283000	0.05	Soft Tissue	
G318R	0.00314000	0.05	Kidney	
L267R	0.00314000	0.05	Liver	
G164S	0.00536000	0.05	Haem and Lymph	
P215H	0.00607000	0.05	Endometrium	
G120D	0.008	0.10	Skin	
R205K	0.0082	0.10	Lung	
A241V	0.01002000	0.10	Lung	rs766238005
L245V	0.01113000	0.10	Lung	
W100L	0.01214000	0.10	Skin	
N155S	0.01295000	0.10	Skin	
G270R	0.01326000	0.10	Lung	
G167D	0.01427000	0.10	Prostate	
P141S	0.01488000	0.10	Lung	
S244C	0.01528000	0.10	Liver	
K146N	0.0164	0.10	Large Intestine	
R218W	0.01791000	0.10	Skin	rs528376420
A310T	0.01893000	0.10	CNS	rs761761542
W126R	0.02054000	0.10	Stomach	
P215S	0.02146000	0.10	Skin	
D222V	0.02257000	0.10	Kidney, Large intestine	
N228Y	0.0248	0.10	Stomach	
S294F	0.0249	0.10	Skin	
R297Q	0.02844000	0.10	Haem and Lymph	rs140793537
R297 *	0.03186000	0.10	Stomach	rs771080849
Y139 *	0.03273000	0.10	Esophagus	
R189L	0.0339	0.10	Skin	
R137 *	0.03489000	0.10	Skin	rs572486802
^4^ Diagram with mutation distribution 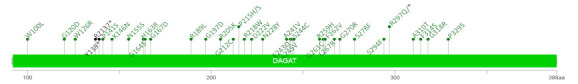				

^1^ Only those mutations with significant VEST values are shown. Mutations are organized by *p*-value significance. ^2^ Significance was chosen for mutations with *p*-value < 0.05 and FDR < 0.1. ^3^ NCBI Identifiers for mutations that have single nucleotide polymorphisms. ^4^ Green labels represent point mutations and black labels represent truncations.

**Table 3:** Expression levels of select DGAT2 mutations.

Sample ID ^1^	Sample Name	Mutation	Mutation Z-Score	Sample Z-Sore Range ^2^
COSU540	Cecum carcinoma	p.G120D	2.221	0.30 ± 4.68
COSU413 ^3^	Bladder urothelial carcinoma	p.P275=	2.335	0.19 ± 1.19
COSU418	Lung squamous cell carcinoma	p.P141S	2.583	0.07 ± 1.18
COSU419	Uterine corpus endometrial carcinoma	p.R189W	2.706	0.22 ± 1.19
COSU540	Cecum carcinoma	p.R218W	3.292	0.30±4.68
COSU418	Lung squamous cell carcinoma	p.W40*	3.368	0.07 ± 1.18
COSU435	Prostate adenocarcinoma	p.G167D	3.647	0.05 ± 1.05
COSU377	Acute myeloid leukemia	c.*27A>G	4.978	0.0040 ± 0.98
COSU414	Breast invasive carcinoma	p.I94V	5.195	0.140 ± 2.34
COSU419	Uterine corpus endometrial carcinoma	c.*556A>T	6.743	0.22 ± 1.19

^1^See Supplementary Figure S3B for tissue information on Sample ID. ^2^ See Supplementary Figure S3A for extended information on Z-score range. ^3^ Red samples are statistically significant.

## Data Availability

All data used in this study i available on COSMIC (https://cancer.sanger.ac.uk/cosmic, accessed on 29 October 2020).

## Supplementary Materials

The following are available online at https://www.mdpi.com/article/10.3390/biology10080714/s1, Figure S1: Sequence alignment of DGAT2 from various animal species, Figure S2: Mutation distribution by age, Figure S3: DGAT2 cohorts expression levels, Table S1: DGAT2 sequences used for the alignment in Supplementary Figure S1, Table S2: DGAT2 mutations reported on COSMIC separated by cancer type, Table S3: CRAVAT analyses, Table S4: Expression data for TCGA samples, Table S5: DGAT2 expression profiles in copy number variants.
