# Supplementary material for: Comprehensive Genetic Analysis of DGAT2 Mutations and Gene Expression Patterns in Human Cancers
Source: Biology (Basel). 2021 Jul 26;10(8):714. doi: 10.3390/biology10080714 (PMC8389207; doi:10.3390/biology10080714)
Supplement: Supplementary file 1 [file biology-10-00714-s001.zip › biology-1273172- supplementary Figures S1-S3_Tables S1 and S2.pdf]

biology

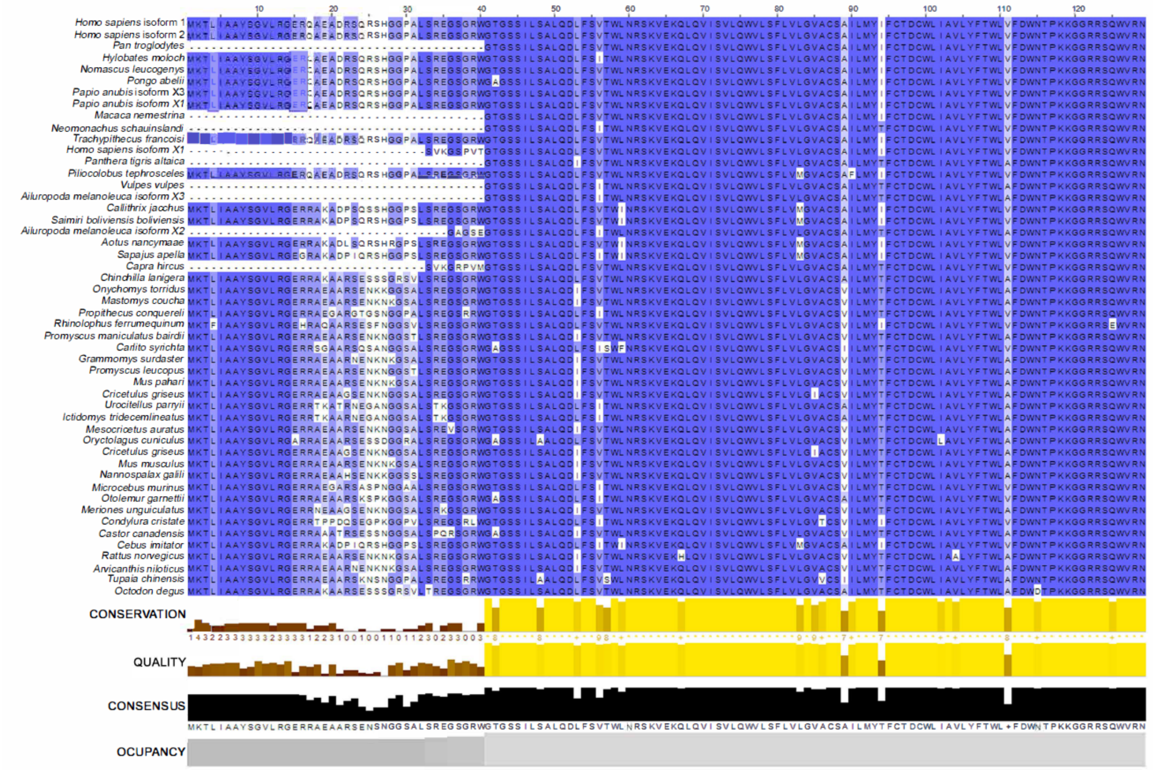

Figure S1. Sequence alignment of DGAT2 from various animal species. Protein alignments were generated using COBALT.

A

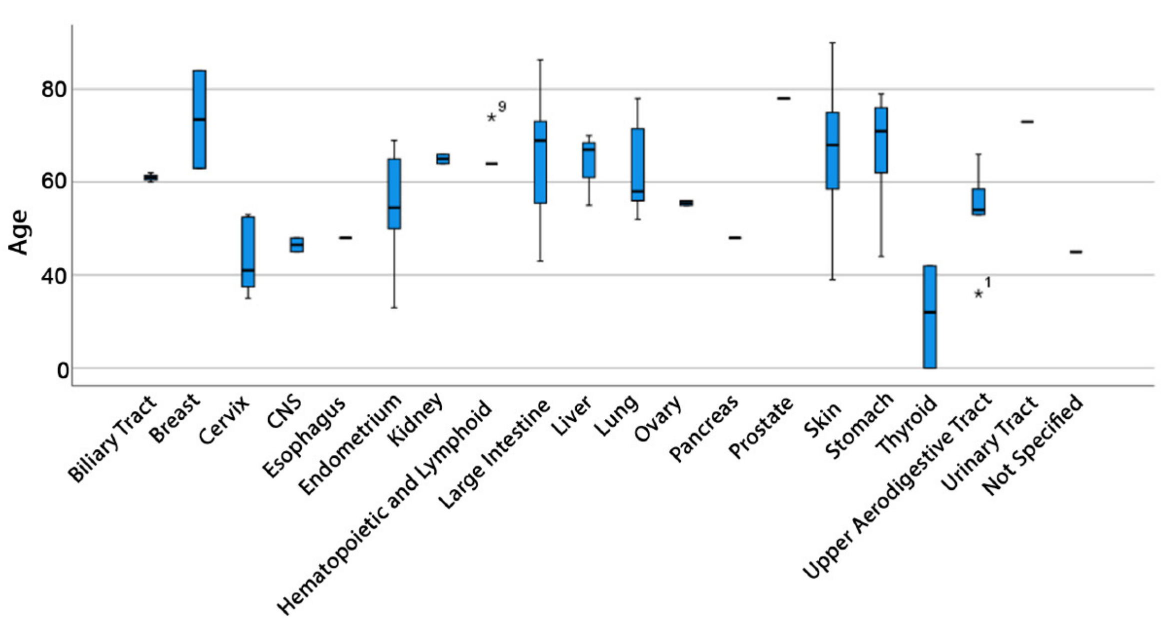

B

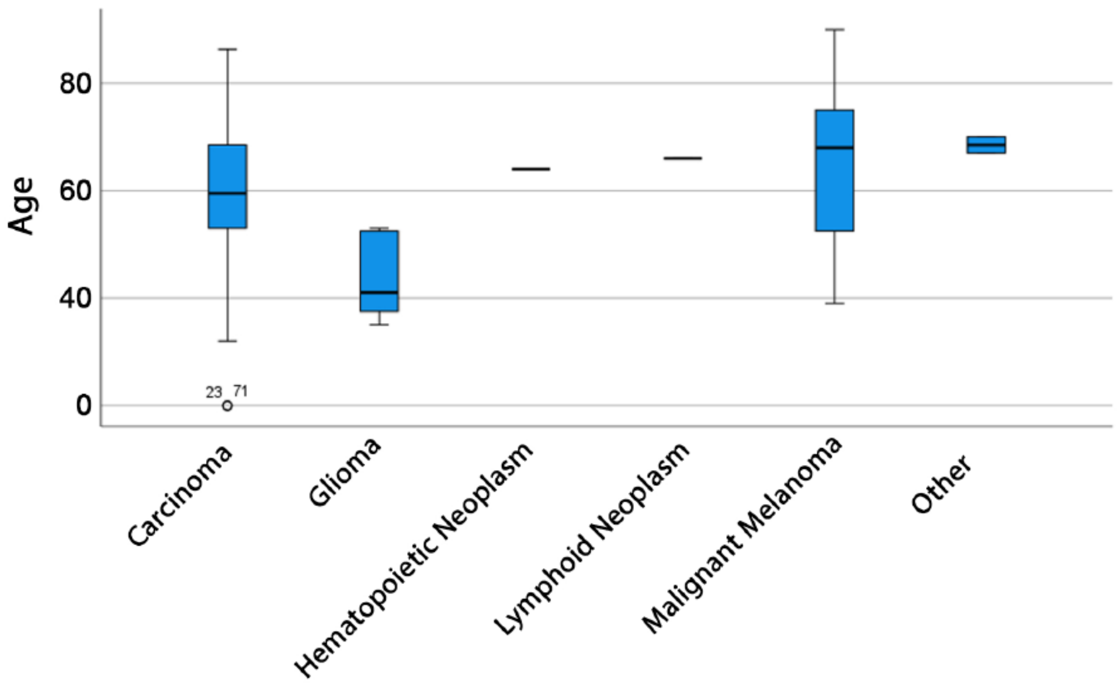

Figure S2. Mutation distribution by age. Extension of Fig. 2 in main text. COSMIC reports for certain samples. Age data was graphed by tissue type (**A**) and cancer type (**B**).

A.

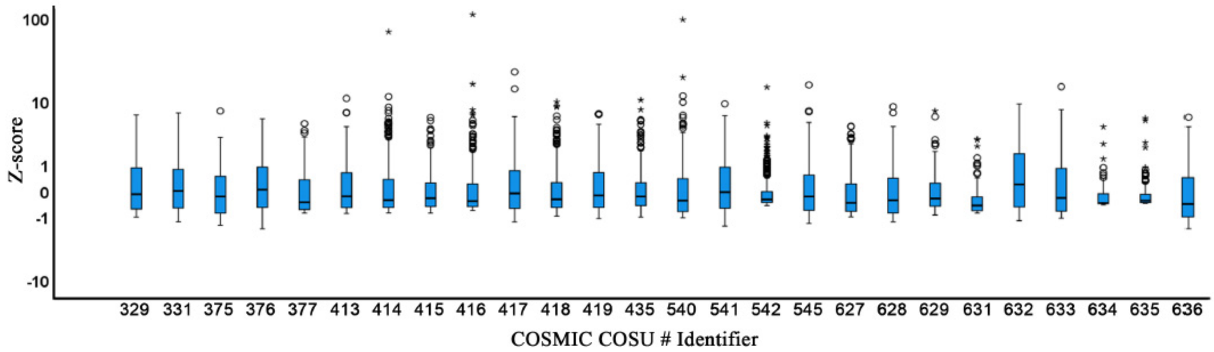

B.

Sample ID	Sample name	Sample ID	Sample name
COSU329	Glioblastoma multiforme	COSU540	Cecum carcinoma
COSU331	Renal cell carcinoma	COSU541	Breast cancer
COSU375	Rectum adenocarcinoma	COSU542	Head and neck thyroid carcinoma
COSU376	Colon adenocarcinoma	COSU545	Brain lower grade glioma
COSU377	Acute myeloid leukemia	COSU627	Head and neck squamous cell carcinoma
COSU413	Bladder urethrial carcinoma	COSU628	Liver hepatocellular carcionma
COSU414	Breast invasive carcinoma	COSU629	Pancreatitc cancer
COSU415	Cervical squamous carcinoma	COSU631	Adenocortical carcinoma
COSU416	Renal cell carcinoma	COSU632	Lymphoid neoplasm difusse large B-cell lymphoma
COSU417	Lung adenocarcinoma	COSU633	Esophageal carcinoma
COSU418	Lung squamous cell carcinoma	COSU634	Kidney chromophobe
COSU419	Uterine corpus endometrial carcinoma	COSU635	Sarcoma
COSU435	Prostate adenocarcionma	COSU636	Uterine carcinocarcinoma

Figure S3. DGAT2 cohorts expression levels. **A.** DGAT2 Z-score distributions for the various cohorts listed on COSMIC. **B.** COSMIC sample ID legend.

Table S1. DGAT2 sequences used for the alignmentin Supplementary Figure S1.

**>NP_115953.2:1-388 diacylglycerol O-acyltransferase 2 isoform 1 [Homo sapiens]**
MKTLIAAYSGVLRGERQAEADRSQRSHGGPALSREGSGRWGTGSSIL-SALQDLFSVTWLNRSKVEKQLQVISVLQWVLSFLVLGVACSAILMYIFCTDCWLIAVLYFTWLVFDWNTPKKGGRRSQWVRNWAVWRYFRDYFPIQLVKTHNLLTTRNYIFGYHPHGIMGLGAFCNFSTEATEV-SKKFPGIRPYLATLAGNFRMPVLREYLMSGGICPVSRDTIDYLLSKNGSGNAIIIVVGGAAESLSSMPGKNAVTLRNRKGFVKLALRHGADLVPIYSFGENEVYKQVIFEEGSWGRWVQKKFQKYIGFAPCIFHGR-GLFSSDTWGLVPYSKPITTVVGEPITIPKLEHPTQQDIDLYHTMYMEALVKLFDKHKTKFGLPETEVLEVN
**>CAD38961.1:47-434 hypothetical protein, partial [Homo sapiens]**
MKTLIAAYSGVLRGERQAEADRSQRSHGGPALSREGSGRWGTGSSIL-SALQDLFSVTWLNRSKVEKQLQVISVLQWVLSFLVLGVACSAILMYIFCTDCWLIAVLYFTWLVFDWNTPKKGGRRSQWVRNWAVWRYFRDYFPIQLVKTHNLLTTRNYIFGYHPHGIMGLGAFCNFSTEATEV-SKKFPGIRPYLATLAGNFRMPVLREYLMSGGICPVSRDTIDYLLSKNGSGNAIIIVVGGAAESLSSMPGKNAVTLRNRKGFVKLALRHGADLVPIYSFGENEVYKQVIFEEGSWGRWVQKKFQKYIGFAPCIFHGR-GLFSSDTWGLVPYSKPITTVVGEPITIPKLEHPTQQDIDLYHTMYMEALVKLFDKHKTKFGLPETEVLEVN
**>XP_009422059.2:25-372 diacylglycerol O-acyltransferase 2 isoform X2 [Pan troglodytes]**
GTGSSILSALQDLFSVTWLNRSKVEKQLQVISVLQWVLSFLVLGVACSAILMYIFCTDCW-LIAVLYFTWLVFDWNTPKKGGRRSQWVRNWAVWRYFRDYFPIQLVKTHNLLTTRNYIFGYHPHGIMGLGAFCNFSTEATEVSKKFPGIRPYLATLAGNFRMPVLREYLMSGGICPVSRDTIDYLLSKNGSG-NAIIIVVGGAAESLSSMPGKNAVTLRNRKGFVKLALRHGADLVPIYSFGENEVYKQVIFEEGSWGRWVQKKFQKYIGFAPCIFHGRGLFSSDTWGLVPYSKPITTVVGEPITIPKLEHPTQQDIDLYHT-MYMEALVKLFDKHKTKFGLPETEVLEVN

**>XP_032022849.1:1-388 diacylglycerol O-acyltransferase 2 [Hylobates moloch]**
MKTLIAAYSGVLRGERQAEADRSQRSHGGPALSREGSGRWGTGSSILSALQDLFSIT-<br>WLNRSKVEKQLQVISVLQWVLSFLVLGVACSAILMYIFCTDCWLIAVLYFTWLVFDWNTPKKGGRRSQWVRNWAV<br>WRYFRDYFPIQLVKTHNLLTTRNYIFGYHPHGIMGLGAFCNFSTEATEV-<br>SKKFPGIRPYLATLAGNFRMPVLREYLMSGGICPVSRDTIDYLLSKNGSGNAIIIVVGGAAESLSSMPGKNAVTLRNR<br>KGFVKLALRHGADLVPIYSFGENEVYKQVIFEEGSWGRWVQKKFQKYIGFAPCIFHGR-<br>GLFSSDTWGLVPYSKPITTVVGEPITIPKLEHPTQQDIDLYHTMYMEALVKLFDKHKTKFGLLETEVLEVN
**>XP_030684545.1:1-388 diacylglycerol O-acyltransferase 2 [Nomascus leucogenys]**
MKTLIAAYSGVLRGERQAEADRSQRSHGGPALSREGSGRWGTGSSIL-<br>SALQDLFSVTWLNRSKVEKQLQVISVLQWVLSFLVLGVACSAILMYIFCTDCWLIAVLYFTWLVFDWNTPKKGGRRS<br>QWVRNWAVWRYFRDYFPIQLVKTHNLLTTRNYIFGYHPHGIMGLGAFCNFSTEATEV-<br>SKKFPGIRPYLATLAGNFRMPVLREYLMSGGICPVSRDTIDYLLSKNGSGNAIIIVVGGAAESLSSMPGKNAVTLRNR<br>KGFVKLALRHGADLVPIYSFGENEVYKQVIFEEGSWGRWVQKKFQKYIGFAPCIFHGR-<br>GLFSSNTWGLVPYSKPITTVVGEPITIPKLEHPTQQDIDLYHTMYMEALVKLFDKHKTKFGLLETEVLEVN
**>XP_002822303.2:1-388 diacylglycerol O-acyltransferase 2 [Pongo abelii]**
MKTLIAAYSGVLRGERQAEADRSQRSHGGPALSREGSGRWGAGSSIL-<br>SALQDLFSVTWLNRSKVEKQLQVISVLQWVLSFLVLGVACSAILMYIFCTDCWLIAVLYFTWLVFDWNTPKKGGRRS<br>QWVRNWAVWRYFRDYFPIQLVKTHNLLTTRNYIFGYHPHGIMGLGAFCNFSTEATEV-<br>SKKFPGIRPYLATLAGNFRMPVLREYLMSGGICPVSRDTIDYLLSKNGSGNAIIIVVGGAAESLSSMPGKNAVTLRNR<br>KGFVKLALRHGADLVPIYSFGENEVYKQVIFEEGSWGRWVQKKFQKYIGFAPCIFHGR-<br>GLFSSDTWGLVPYSKPITTVVGEPITIPKLEHPTQQDIDLYHTMYMEALVKLFDKHKTKFGLLETEVLEVN
**>XP_003910483.1:1-388 diacylglycerol O-acyltransferase 2 isoform X3 [Papio anubis]**
MKTLIAAYSGVLRGERQAEADRSQRSHGGPALSREGSGRWGTGSSIL-<br>SALQDLFSVTWLNRSKVEKQLQVISVLQWVLSFLVLGVACSAILMYIFCTDCWLIAVLYFTWLVFDWNTPKKGGRRS<br>QWVRNWAVWRYFRDYFPIQLVKTHNLLTTRNYIFGYHPHGIMGLGAFCNFSTEATEV-<br>SKKFPGIRPYLATLAGNFRLPVLREYLMS
GGICPVNRDTIDYLLSKNGSGNAIIIVVGGAAESLSSMPGKNAVTLRN-<br>RKGFVKLALRHGADLVPMYSFGENEVYKQVIFEEGSWGRWVQKKFQKYIGFAPCIFHGRGLFSSDTWGLVPYSKPIT<br>TVVGEPITIPKLEHPTQQDIDLYHTMYMEALVKLFDKHKTKFGLLETEVLEVN
**>XP_021782024.1:1-387 diacylglycerol O-acyltransferase 2 isoform X1 [Papio anubis]**
MKTLIAAYSGVLRGERQAEADRSQRSHGGPALSREGSGRWGTGSSIL-<br>SALQDLFSVTWLNRSKVEKQLQVISVLQWVLSFLVLGVACSAILMYIFCTDCWLIAVLYFTWLVFDWNTPKKGGRRS<br>QWVRNWAVWRYFRDYFPIQLVKTHNLLTTRNYIFGYHPHGIMGLGAFCNFSTEATEV-<br>SKKFPGIRPYLATLAGNFRLPVLREYLMSGGICPVNRDTIDYLLSKNGSGNAIIIVVGGAAESLSSMPGKNAVTLRNRK<br>GFVKLALRHGADLVPMYSFGENEVYKQVIFEEGSWGRWVQKKFQKYIGFAPCIFHGR-<br>GLFSSDTWGLVPYSKPITTVVGEPITIPKLEHPTQQDIDLYHTMYMEALVKLFDKHKTKFGLLETEVLEV
**>XP_011717414.1:31-378 diacylglycerol O-acyltransferase 2 isoform X2 [Macaca nemestrina]**
GTGSSILSALQDLFSVTWLNRSKVEKQLQVISVLQWVLSFLVLGVACSAILMYIFCTDCW-<br>LIAVLYFTWLVFDWNTPKKGGRRSQWVRNWAVWRYFRDYFPIQLVKTHNLLTTRNYIFGYHPHGIMGLGAFCNFS<br>TEATEVSKKFPGIRPYLATLAGNFRLPVLREYLMSGGICPVNRDTIDYLLSKNGSG-<br>NAIIIVVGGAAESLSSMPGKNAVTLRNRKGFVKLALRHGADLVPMYSFGENEVYKQVIFEEGSWGRWVQKKFQKYI<br>GFAPCIFHGRGLFSSDTWGLVPYSKPITTVVGEPITIPKLEHPTQQDIDLYHT-<br>MYMEALVKLFDKHKTKFGLLETEVLEVN
**>XP_021560351.1:2-349 diacylglycerol O-acyltransferase 2 isoform X2 [Neomonachus schauinslandi]**
GTGSSILSALQDLFSITWLNRSKVEKQLQVISVLQWVLSFLVLGVACSAILMYTFCTDCW-<br>LIAVLYFTWLVFDWNTPKKGGRRSQWVRNWAVWRYFRDYFPIQLVKTHNLLTTRNYIFGYHPHGIMGLGAFCNFS<br>TEATEVSKKFPGIRPYLATLAGNFRMPVLREYLMSGGICPVNRDTIDYLLSKNGSG-<br>NAIIIVVGGAAESLSSMPGKNAVTLRNRKGFVKLALRHGADLVPTYSFGENEVYKQVIFEEGSWGRWVQKKFQKYI<br>GFAPCIFHGRGLFSSDTWGLVPYSKPITTVVGEPITIPKLEHPTQQDIDLYHT-<br>MYMEALVKLFDKHKTKFGLPETEVLEVN

>XP_033059700.1:1-388 LOW QUALITY PROTEIN: diacylglycerol O-acyltransferase 2 [Trachypithecus francoisi]

MKTLIAAYSGVLRGERQAEADRSQRSHGGPALSREGSGRWGTGSSIL-
SALQDLFSVTWLNRSKVEKQLQVISVLQWVLSFLVLGVACSAILMYIFCTDCWLIAVLYFTWLVFDWNTPKKGGRRS
QWVRNWAVWRYFRDYFPIQLVKTHNLLTXRNYIFGYHPHGIMGLGAFCNFSTEATEV-
SKKFPGIRPYLATLAGNFRLPVLREYLMSGGICPVNRDTIDYLLSKNGSGNAIIIVVGGAAESLSSMPGKNAVTLRNRK
GFVKLALRHGADLVPMYSFGENEVYKQVIFEEGSWGRWVQKKFQKYIGFAPCIFHGR-
GLFSSDTWGLVPYSKPITTVVGEPITIPKLEHPTQQDIDLYHTMYMEALVKLFDKHKTKFGLLETEVLEVN

>XP_011543606.1:3-358 diacylglycerol O-acyltransferase 2 isoform X1 [Homo sapiens]

SVKGSPVTGTGSSILSALQDLFSVTWLNRSKVEKQLQVIS-
VLQWVLSFLVLGVACSAILMYIFCTDCWLIAVLYFTWLVFDWNTPKKGGRRSQWVRNWAVWRYFRDYFPIQLVKT
HNLLTTRNYIFGYHPHGIMGLGAFCNFSTEATEVSKKFPGIRPYLATLAGNFRM-
PVLREYLMSGGICPVSRDTIDYLLSKNGSGNAIIIVVGGAAESLSSMPGKNAVTLRNRKGFVKLALRHGADLVPIYSF
GENEVYKQVIFEEGSWGRWVQKKFQKYIGFAPCIFHGR-
GLFSSDTWGLVPYSKPITTVVGEPITIPKLEHPTQQDIDLYHTMYMEALVKLFDKHKTKFGLPETEVLEVN

>XP_007080981.1:7-354 PREDICTED: diacylglycerol O-acyltransferase 2, partial [Panthera tigris altaica]

GTGSSILSALQDIFSVTWLNRSKVEKQLQVISVLQWVLSFLVLGVACSAILMYTFCTDCW-
LIAVLYFTWLAFDWNTPKKGGRRSQWVRNWAVWRYFRDYFPIQLVKTHNLLTTRNYIFGYHPHGIMGLGAFCNFS
TEATEVSKKFPGIRPYLATLAGNFRMPVLREYLMSGGICPVNRDTIDYLLSKNGSG-
NAIIIVVGGAAESLSSMPGKNAVTLRNRKGFVKLALRHGADLVPIYSFGENEVYKQVIFEEGSWGRWVQKKFQKYIG
FAPCIFHGRGLFSSDTWGLVPYSKPITTVVGEPITIPKLEHPTQQDID-
LYHSMYMEALVKLFDKHKTKFGLPETEVLEVN **>XP_023065138.1:1-388 diacylglycerol O-acyltransferase 2 [Piliocolobus tephrosceles]**

MKTLIAAYSGVLRGERQAEADRSQRSHGGPALSREGSGRWGTGSSIL-
SALQDLFSVTWLNRSKVEKQLQVISVLQWVLSFLVMGVACSAFLMYIFCTDCWLIAVLYFTWLVFDWNTPKKGGRR
SQWVRNWAVWRYFRDYFPIQLVKTHNLLTTRNYIFGYHPHGIMGLGAFCNFSTEATEV-
SKKFPGIRPYLATLAGNFRLPVLREYLMSGGICPVNRDTIDYLLSKNGSGNAIIIVVGGAAESLSSMPGKNAVTLRNRK
GFVKLALRHGADLVPMYSFGENEVYKQVIFEEGSWGRWVQKKFQKYIGFAPCIFHGR-
GLFSSDTWGLVPYSKPITTVVGEPITIPKLEHPTQQDIDLYHTMYMEALVKLFDKHKTKFGLLETEVLEVN

>XP_025866396.1:223-570 diacylglycerol O-acyltransferase 2 [Vulpes vulpes]

GTGSSILSALQDLFSITWLNRSKVEKQLQVISVLQWVLSFLVLGVACSAILMYTFCTDCW-
LIAVLYFTWLAFDWNTPKKGGRRSQWVRNWAVWRYFRDYFPIQLVKTHNLLTTRNYIFGYHPHGIMGLGAFCNFS
TEATEVSKKFPGIRPYLATLAGNFRMPVLREYLMSGGICPVNRDTIDYLLSKNGSG-
NAIIIVVGGAAESLSSMPGKNAVTLRNRKGFVKLALRHGADLVPTYSFGENEVYKQVIFEEGSWGRWVQKKFQKYI
GFAPCIFHGRGLFSSDTWGLVPYSKPITTVVGEPITIPKLEHPTQQDID-
LYHAMYMEALVKLFDKHKTKFGLPETEVLEVN

>XP_034522208.1:23-370 diacylglycerol O-acyltransferase 2 isoform X3 [Ailuropoda melanoleuca]

GTGSSILSALQDLFSITWLNRSKVEKQLQVISVLQWVLSFLVLGVACSAILMYTFCTDCW-
LIAVLYFTWLAFDWNTPKKGGRRSQWVRNWAVWRYFRDYFPIQLVKTHNLLTTRNYIFGYHPHGIMGLGAFCNFS
TEATEVSKKFPGIRPYLATLAGNFRMPVLREYLMSGGICPVNRDTIDYLLSKNGSG-
NAIIIVVGGAAESLSSMPGKNAVTLRNRKGFVKLALRHGADLVPTYSFGENEVYKQVIFEEGSWGRWVQKKFQKYI
GFAPCIFHGRGLFSSDTWGLVPYSKPITTVVGEPITIPKLEHPTQQDID-
LYHAMYMEALVKLFDKHKTKFGLPETEVLEVN

>XP_009005662.2:1-388 diacylglycerol O-acyltransferase 2 [Callithrix jacchus]

MKTLIAAYSGVLRGERRAKADPSQSSHGGPSLSREGSGRWGTGSSIL-
SALQDLFSVTWINRSKVEKQLQVISVLQWVLSFLVMGVACSAILMYIFCTDCWLIAVLYFTWLVFDWNTPKKGGRRS
QWVRNWAVWRYFRDYFPIQLVKTHNLSTTRNYIFGYHPHGIMGLGAFCNFSTEATEV-
SKKFPGIRPYLATLAGNFRMPVLREYLMSGGICPVNRDTIDYLLSKNGSGNAIIIVVGGAAESLSSMPGKNAVTLRNR
KGFVKLALRHGADLVPIYSFGENEVYKQVIFEEGSWGRWVQKKFQKYIGFAPCIFHGR-
GLFSSDTWGLVPYSKPITTVVGEPITIPKLEHPTQQDIDLYHTMYMEALVKLFDKHKTKFGLPETEVLEVN

>XP_003923533.1:1-388 diacylglycerol O-acyltransferase 2 [Saimiri boliviensis boliviensis]

MKTLIAAYSGVLRGERRAKADPSQRSHGGPSLSREGSGRWGTGSSIL-<br>SALQDLFSVTWINRSKVEKQLQVISVLQWVLSFLVMGVACSAILMYIFCTDCWLIAVLYFTWLVFDWNTPKKGGRRS<br>QWVRNWAVWRYFRDYFPIQLVKTHNLPTTRNYIFGYHPHGIMGLGAFCNFSTEATEV-<br>SKKFPGIRPYLATLAGNFRMPVLREYLMSGGICPVNRDTIDYLLSKNGSGNAIIIVVGGAAESLSSMPGKNAVTLRNR<br>KGFVKLALRHGADLVPIYSFGENEVYKQVIFEEGSWGRWVQKKFQKYIGFAPCIFHGR-<br>GLFSSDTWGLVPYSKPITTVVGEPITIPKLEHPTQQDIDLYHTMYMEALVKLFDKHKTKFGLPESEVLEVN
**>XP_034522207.1:19-371 diacylglycerol O-acyltransferase 2 isoform X2 [Ailuropoda melanoleuca]**
GAGSEGTGSSILSALQDLFSITWLNRSKVEKQLQVIS-<br>VLQWVLSFLVLGVACSAILMYTFCTDCWLIAVLYFTWLAFDWNTPKKGGRRSQWVRNWAVWRYFRDYFPIQLVKT<br>HNLLTTRNYIFGYHPHGIMGLGAFCNFSTEATEVSKKFPGIRPYLATLAGNFRM-<br>PVLREYLMSGGICPVNRDTIDYLLSKNGSGNAIIIVVGGAAESLSSMPGKNAVTLRNRKGFVKLALRHGADLVPTYSF<br>GENEVYKQVIFEEGSWGRWVQKKFQKYIGFAPCIFHGR-<br>GLFSSDTWGLVPYSKPITTVVGEPITIPKLEHPTQQDIDLYHAMYMEALVKLFDKHKTKFGLPETEVLEVN
**>XP_012325065.1:1-388 diacylglycerol O-acyltransferase 2 isoform X1 [Aotus nancymaae]**
MKTLIAAYSGVLRGERRAKADLSQRSHRGPSLSREGSGRWGTGSSIL-<br>SALQDLFSVTWINRSKVEKQLQVISVLQWVLSFLVMGVACSAILMYIFCTDCWLIAVLYFTWLVFDWNTPKKGGRRS<br>QWVRNWAVWRYFRDYFPIQLVKTHNLPTTRNYIFGYHPHGIMGLGAFCNFSTEATEV-<br>SKKFPGIRPYLATLAGNFRMPVLREYLMSGGICPVNRDTIDYLLSKNGSGNAIIIVVGGAAESLSSMPGKNAVTLRNR<br>KGFVKLALRHGADLVPTYSFGE<br>NEVYKQVIFEEGSWGRWVQKKFQKYIGFAPCIFHGR-<br>GLFSSDTWGLVPYSKPITTVVGEPITIPKLEHPTQQDIDLYHTMYMEALVKLFDKHKTKFGLPETEVLEVN
**>XP_032114997.1:1-388 diacylglycerol O-acyltransferase 2 [Sapajus apella]**
MKTLIAAYSGVLRGEGRAKADPIQRSHGGPSLSREGSGRWGTGSSILSALQDLFSIT-<br>WINRSKVEKQLQVISVLQWVLSFLVMGVACSAILMYIFCTDCWLIAVLYFTWLVFDWNTPKKGGRRSQWVRNWAV<br>WRYFRDYFPIQLVKTHNLSTTRNYIFGYHPHGIMGLGAFCNFSTEATEV-<br>SKKFPGIRPYLATLAGNFRMPVLREYLMSGGICPVNRDTIDYLLSKNGSGNAIIIVVGGAAESLSSMPGKNAVTLRNR<br>KGFVKLALRHGADLVPIYSFGENEVYKQVIFEEGSWGRWVQKKFQKYIGFAPCIFHGR-<br>GLFSSDTWGLVPYSKPITTVVGEPITIPKLEHPTQQDIDLYHTMYMEALVKLFDKHKTKFGLPETEVLEVN
**>XP_017914342.1:3-358 PREDICTED: diacylglycerol O-acyltransferase 2 isoform X1 [Capra hircus]**
SVKGRPVMGTGSSILSALQDLFSVTWLNRSKVEKQLQVIS-<br>VLQWVLSFLVLGVACSAILMYTFCTDCWLIAVLYFTWLVFDWNTPKKGGRRSQWVRNWAVWRYFRDYFPIQLVKT<br>HNLLTSRNYIFGYHPHGIMGLGAFCNFSTEATEVSKKFPGIRPYLATLAGNFRM-<br>PVLREYLMSGGICPVNRDTIDYLLSKNGSGNAIIIVVGGAAESLSSMPGKNAVTLRNRKGFVKLALRHGADLVPIYSF<br>GENEVYKQVIFEEGSWGRWVQKKFQKYIGFAPCIFHGR-<br>GLFSSDTWGLVPYSKPITTVVGEPITIPKLEHPTQQDIDLYHAMYMEALVKLFDQHKTKFGLPETEVLEVN
**>XP_005379934.1:1-388 PREDICTED: diacylglycerol O-acyltransferase 2 isoform X1 [Chinchilla lanigera]**
MKTLIAAYSGVLRGERRAKAARSESSSGRSVLSREGSGRWGTGSSIL-<br>SALQDLFSVTWLNRSKVEKQLQVISVLQWVLSFLVLGVACSAILMYTFCTDCWLIAVLYFTWLAFDWNTPKKGGRR<br>SQWVRNWAVWRYFRDYFPIQLVKTHNLPTTRNYIFGYHPHGIMGLGAFCNFSTEATEV-<br>SKKFPGIRPYLATLAGNFRMPVLREYLMSGGICPVNRDTIDYLLSKNGSGNAIIIVVGGAAESLSSMPGKNAVTLKNR<br>KGFVKLALRHGADLVPIYSFGENEVYKQVIFEEGSWGRWVQKKFQKYIGFAPCIFHGR-<br>GLFSSDTWGLVPYSKPITTVVGEPITIPKVEHPTQQDIDLYHSMYMEALVKLFDKHKTKFGLPETEVLEVN
**>XP_036031633.1:1-388 diacylglycerol O-acyltransferase 2 [Onychomys torridus]**
MKTLIAAYSGVLRGERRAEAARSENKKGGSALSREGSGRWGTGSSIL-<br>SALQDIFSVTWLNRSKVEKQLQVISVLQWVLSFLVLGVACSVILMYTFCTDCWLIAVLYFTWLAFDWNTPKKGGRRS<br>QWVRNWAVWRYFRDYFPIQLVKTHNLLTTRNYIFGYHPHGIMGLGAFCNFSTEATEV-<br>SKKFPGIRPYLATLAGNFRMPVLREYLMSGGICPVNRDTIDYLLSKNGSGNAIIIVVGGAAESLSSMPGKNAVTLRNR<br>KGFVKLALRHGADLVPTYSFGENEVYKQVIFEEGSWGRWVQKKFQKYIGFAPCIFHGR-<br>GLFSSDTWGLVPYSKPITTVVGEPITIPKMEHPTQKDIDLYHAMYMEALVKLFDNHKTKFGLPETEVLEVN
**>XP_031243344.1:1-388 diacylglycerol O-acyltransferase 2 isoform X1 [Mastomys coucha]**

MKTLIAAYSGVLRGERRAEAARSENKNKGSALSREGSGRWGTGSSIL-<br>SALQDIFSVTWLNRSKVEKQLQVISVLQWVLSFLVLGVACSVILMYTFCTDCWLIAVLYFTWLAFDWNTPKKGGRRS<br>QWVRNWAVWRYFRDYFPIQLVKTHNLLTTRNYIFGYHPHGIMGLGAFCNFSTEATEV-<br>SKKFPGIRPYLATLAGNFRMPVLREYLMSGGICPVNRDTIDYLLSKNGSGNAIIIVVGGAAESLSSMPGKNAVTLRNR<br>KGFVKLALRHGADLVPTYSFGENEVYKQVIFEEGSWGRWVQKKFQKYIGFAPCIFHGR-<br>GLFSSDTWGLVPYSKPITTVVGEPITVPKLEHPTQKDIDLYHTMYMEALVKLFDNHKTKFGLPETEVLEVN
**>XP_012501204.1:1-388 PREDICTED: diacylglycerol O-acyltransferase 2 [Propithecus coquereli]**
MKTLIAAYSGVLRGERRAEGARGTGSNGGPALSREGSRRWGTGSSILSALQDLFSIT-<br>WLNRSKVEKQLQVISVLQWVLSFLVLGVACSVILMYTFCTDCWLIAVLYFTWLVFDWNTPKKGGRRSQWVRNWA<br>VWRYFRDYFPIQLVKTHNLLTTRNYIFGYHPHGIMGLGAFCNFSTEATEV-<br>SKKFPGIRPYLATLAGNFRMPVLREYLMSGGICPVNRDTIDYLLSKNGSGNAIIIVVGGAAESLSSMPGKNAVTLRNR<br>KGFVKLALRHGADLVPTYSFGENEVYKQVIFQEGSWGRWVQKKFQKYIGFAP-<br>CIFHGRGFFSSDTWGLVPYSKPITTVVGEPITIPKLEHPTQQDIDLYHAMYMEALVKLFDNHKTKFGLPETEVLEVN
**>KAF6332887.1:1-388 diacylglycerol O-acyltransferase 2 [Rhinolophus ferrumequinum]**
MKTFIAAYSGVLRGEHRAQAARSESFNGGSVLSREGSGRWGTGSSIL-<br>SALQDLFSVTWLNRSKVEKQLQVISVLQWVLSFLVLGVACSVILMYIFCTDCWLIAVLYFTWLVFDWNTPKKGGRRS<br>EWVRNWAVWRYFRDYFPIQLVKTHNLLTTRNYIFGYHPHGIMGLGAFCNFSTEATEV-<br>SKKFPGIRPYLATLAGNFRMPVLREYLMSGGICPVNRDTIDYLLSKNGSGNAIIIVVGGAAESLSSRPGKNAVTLRNR<br>KGFVKLALRHGADLVPTYSFGENEVYKQVIFEEGSWGRWVQKKFQKYIGFAPCIFHGR-<br>GLFSSDTWGLVPYSKPITTVVGEPITIPKLEHPTQQDIDLYHAMYMEALVKLFDKHKTKFGLPETEVLEVN
**>XP_006982228.1:1-388 PREDICTED: diacylglycerol O-acyltransferase 2 isoform X1 [Peromyscus maniculatus bairdii]**
MKTLIAAYSGVLRGERRAEAARSENKNGGSTLSREGSGRWGTGSSIL-<br>SALQDIFSVTWLNRSKVEKQLQVISVLQWVLSFLVLGVACSVILMYTFCTDCWLIAVLYFTWLAFDWNTPKKGGRRS<br>QWVRNWAVWRYFRDYFPIQLVKTHNLLTTRNYIFGYHPHGIMGLGAFCNFSTEATEV-<br>SKKFPGIRPYLATLAGNFRMPVLREYLMSGGICPVNRDTIDYLLSKNGSGNAIIIVVGGAAESLSSMPGKNAVTLRNR<br>KGFVKLALRHGADLVPTYSFGENEVYKQVIFEEGSWGRWVQKKFQKYIGFAPCIFHGR-<br>GLFSSDTWGLVPYSKPITTVVGEPITIPKMEHPTQKDIDLYHAMYMEALVKLFDNHKTKFGLPETEVLEVN
**>XP_008056012.1:1-388 diacylglycerol O-acyltransferase 2 isoform X1 [Carlito syrichta]**
MKTLIAAYSGVLRGERRSGAARSQSANGGSALSREGSGRWGAGSSIL-<br>SALQDLFSISWFNRSKVEKQLQVISVLQWVLSFLVLGVACSIILMYTFCTDCWLIAVLYFTWLVFDWNTPKKGGRRSQ<br>WVRNWAVWRYFRDYFPIQLVKTHNLLTTRNYIFGYHPHGIMGLGAFCNFSTEATEV-<br>SKKFPGIRPYLATLAGNFRMPVLREYLMSGGICPVNRDTIDYLLSKNGNGNAIIIVVGGAAESLSSMPGKNAVTLRNR<br>KGFVKLALRHGADLVPVYSFGENEVYKQVIFEEGSWGRWVQKKFQKYIGFAPCIFHGR-<br>GLFSSDTWGLVPYSKPITTVVGEPITIPKLEHPTQQDIDLYHAMYMEALVKLFDKHKTKFGLPETEVLEVN
**>XP_028626471.1:1-388 diacylglycerol O-acyltransferase 2 [Grammomys surdaster]**
MKTLIAAYSGVLRGERRAEAARNENKNKGSALSREGSGRWGTGSSIL-<br>SALQDIFSVTWLNRSKVEKQLQVISVLQWVLSFLVLGVACSVILMYTFCTDCWLIAVLYFTWLAFDWNTPKKGGRRS<br>QWVRNWAVWRYFRDYFPIQLVKTHNLLTTRNYIFGYHPHGIMGLGAFCNFSTEATEV-<br>SKKFPGIRPYLATLAGNFRMPVLREYLMSGGICPVNRDTIDYLLSKNGSGNAIIIVVGGAAESLSSMPGKNAVTLRNR<br>KGFVKLALRHGADLVPTYSFGENEVYKQVIFEEGSWGRWVQKKFQKYIGFAPCIFHGR-<br>GLFSSDTWGLVPYSKPITTVVGEPITVPKLEHPTQKDIDLYHTMYMEALVKLFDNHKTKFGLPETEVLEVN
**>XP_028733324.1:1-388 diacylglycerol O-acyltransferase 2 isoform X2 [Peromyscus leucopus]**
MKTLIAAYSGVLRGERRAEAARSENKNGGSTLSREGSGRWGTGSSIL-<br>SALQDIFSVTWLNRSKVEKQLQVISVLQWVLSFLVLGVACSVILMYTFCTDCWLIAVLYFTWLAFDWNTPKKGGRRS<br>QWVRNWAVWRYFRDYFPIQLVKTHNLLTTRNYIFGYHPHGIMGLGAFCNFSTEATEV-<br>SKKFPGIRPYLATLAGNFRMPVLREYLMSGGICPVNRDTIDYLLSKNGSGNAIIIVVGGAAESLSSMPGKNAVTLRNR<br>KGFVKLALRHGADLVPTYSFGENEVYKQVIFEEGSWGRWVQKKFQKYIGFAPCIFHGR-<br>GLFSSDTWGLVPYSKPITTVVGEPITIPKVEHPTQKDIDLYHAMYMEALVKLFDNHKTKFGLPETEVLEVN
**>XP_021062496.1:1-388 diacylglycerol O-acyltransferase 2 [Mus pahari]**

MKTLIAAYSGVLRGERRAEAARSENKNKGSALSREGSGRWGTGSSIL-<br>SALQDIFSVTWLNRSKVEKQLQVISVLQWVLSFLVLGVACSVILMYTFCTDCWLIAVLYFTWLAFDWNTPKKGGRRS<br>QWVRNWAVWRYFRDYFPIQLVKTHNLLTTRNYIFGYHPHGIMGLGAFCNFSTEATEV-<br>SKKFPGIRPYLATLAGNFRMPVLREYLMSGGICPVNRDTIDYLLSKNGSGNAIIIVVGGAAESLSSMPGKNAVTLRNR<br>KGFVKLALRHGADLVPTYSFGENEVYKQVIFEEGSWGRWVQKKFQKYIGFAPCIFHGR-<br>GLFSSDTWGLVPYSKPITTVVGEPITVPKLEHPTQKDIDLYHAMYMEALVKLFDNHKTKFGLPETEVLEVN
**>XP_003507132.1:1-388 diacylglycerol O-acyltransferase 2 [Cricetulus griseus]**
MKTLIAAYSGVLRGERRAEAAGSENKNGGSALSREGSGRWGTGSSIL-<br>SALQDIFSVTWLNRSKVEKQLQVISVLQWVLSFLVLGIACSVILMYTFCTDCWLIAVLYFTWLAFDWNTPKKGGRRS<br>QWVRNWAVWRYFRDYFPIQLVKTHNLLTTRNYIFGYHPHGIMGLGAFCNFSTEATEV-<br>SKKFPGIRPYLATLAGNFRMPVLREYLMSGGICPVNRDTIDYLLSKNGSGNAIIIVVGGAAESLSSMPGKNAVTLRNR<br>KGFVKLALRHGADLVPTYSFGENEVYKQVIFEEGSWGRWVQKKFQKYIGFAPCIFHGR-<br>GLFSSDTWGLVPYSKPITTVVGEPITIPKLEHPTQKDIDLYHAMYMEALVKLFDNHKTKFGLPETEVLEVN
**>XP_026243076.1:1-388 diacylglycerol O-acyltransferase 2 [Urocitellus parryii]**
MKTLIAAYSGVLRGERRTKATRNEGANGGSALSTKGSGRWGTGSSILSALQDLFSIT-<br>WLNRSKVEKQLQVISVLQWVLSFLVLGVACSAILMYTFCTDCWLIAVLYFTWLAFDWNTPKKGGRRSQWVRNWA<br>VWRYFRDYFPIQLVKTHNLLTTRNYIFGYHPHGIMGLGAFCNFSTEATEV-<br>SKKFPGIRPYLATLAGNFRMPVLREYLMSGGICPVNRDTIDYLLSKNGSGNAIIIVVGGAAESLSSMPGKNAVTLRNR<br>KGFVKLALRHGADLVPTYSFGENEVYKQVIFEEGSWGRWVQKKFQKYIGFAPCIFHGR-<br>GLFSSDTWGLVPYSKPITTVVGEPITIPKLEHPTQQDIDLYHAMYMEALVKLFDKHKTKFGLPETEVLEVN
**>XP_005323271.1:1-388 diacylglycerol O-acyltransferase 2 [Ictidomys tridecemlineatus]**
MKTLIAAYSGVLRGERRTKAARNEGANGGSALSTKGSGRWGTGSSILSALQDLFSIT-<br>WLNRSKVEKQLQVISVLQWVLSFLVLGVACSAILMYTFCTDCWLIAVLYFTWLAFDWNTPKKGGRRSQWVRNWA<br>VWRYFRDYFPIQLVKTHNLLTTRNYIFGYHPHGIMGLGAFCNFSTEATEV-<br>SKKFPGIRPYLATLAGNFRMPVLREYLMSGGICPVNRDTIDYLLSKNGSGNAIIIVVGGAAESLSSMPGKNAVTLRNR<br>KGFVKLALRHGADLVPTYSFGENEVYKQVIFEEGSWGRWVQKKFQKYIGFAPCIFHGR-<br>GLFSSDTWGLVPYSKPITTVVGEPITIPKLEHPTQQDIDLYHAMYMEALVKLFDKHKTKFGLPETEVLEVN
**>XP_005074030.1:1-388 diacylglycerol O-acyltransferase 2 [Mesocricetus auratus]**
MKTLIAAYSGVLRGERRAEAARSENKNGGSALSREVSGRWGTGSSIL-<br>SALQDIFSVTWLNRSKVEKQLQVISVLQWVLSFLVLGVACSVILMYTFCTDCWLIAVLYFTWLAFDWNTPKKGGRRS<br>QWVRNWAVWRYFRDYFPIQLVKTHNLLTTRNYIFGYHPHGIMGLGAFCNFSTEATEV-<br>SKKFPGIRPYLATLAGNFRMPVLREYLMSGGICPVNRDTIDYLLSKNGSGNAIIIVVGGAAESLSSMPGKNAVTLRNR<br>KGFVKLALRHGADLVPTYSFGENEVYKQVIFEEGSWGRWVQKKFQKYIGFAPCIFHGR-<br>GLFSSDTWGLVPYSKPITTVVGEPITIPKLEHPTQKDIDLYHAMYMEALVKLFDNHKTKFGLPESEVLEVN
**>XP_008262050.1:1-388 PREDICTED: LOW QUALITY PROTEIN: diacylglycerol O-acyltransferase 2 [Oryctolagus cuniculus]**
MKTLIAAYSGVLRGARRAEAARSESSDGGRALSREGSGRWGAGSSI-<br>LAALQDLFSVTWLNRSKVEKQLQVISVLQWVLSFLVLGVACSVILMYTFCTDCWLLAVLYFTWLAFDWNTPKKGG<br>RRSQWVRNWAVWRYFRDYFPIQLVKTHNLLTTRNYIFGYHPHGIMGLGAFCNFSTEATEV-<br>SKKFPGIRPYLATLAGNFRMPVLREYLMSGGICPVNRDTIDYLLSKNGSGNAIIIVVGGAAESLSSMPGKNAVTLRNR<br>KGFVKLALRHGADLVPTYSFGENEVYKQVIFEEGSWGRWVQKKFQKYIGFAPCIFHGRGLF-<br>SPDTWGLVPYSKPITTVVGEPITIPKLEHPTQQDIDLYHAMYMEALVKLFDKHKTKFGLPESEVLEVN
**>ERE78233.1:1-386 diacylglycerol O-acyltransferase 2 [Cricetulus griseus]**
MKTLIAAYSGVLRGERRAEAAGSENKNGGSALSREGSGRWGTGSSIL-<br>SALQDIFSVTWLNRSKVEKQLQVISVLQWVLSFLVLGIACSVILMYTFCTDCWLIAVLYFTWLAFDWNTPKKGGRRS<br>QWVRNWAVWRYFRDYFPIQLVKTHNLLTTRNYIFGYHPHGIMGLGAFCNFSTEATEV-<br>SKKFPGIRPYLATLAGNFRMPVLREYLMSGGICPVNRDTIDYLLSKNGSGNAIIIVVGGAAESLSSMPGKNAVTLRNR<br>KGFVKLALRHGADLVPTYSFGENEVYKQVIFEEGSWGRWVQKKFQKYIGFAPCIFHGR-<br>GLFSSDTWGLVPYSKPITTVVGEPITIPKLEHPTQKDIDLYHAMYMEALVKLFDNHKTKFGLPETEVLE
**>NP_080660.1:1-388 diacylglycerol O-acyltransferase 2 [Mus musculus]**

MKTLIAAYSGVLRGERRAEAARSENKNKGSALSREGSGRWGTGSSIL-<br>SALQDIFSVTWLNRSKVEKQLQVISVLQWVLSFLVLGVACSVILMYTFCTDCWLIAVLYFTWLAFDWNTPKKGGRRS<br>QWVRNWAVWRYFRDYFPIQLVKTHNLLTTRNYIFGYHPHGIMGLGAFCNFSTEATEV-<br>SKKFPGIRPYLATLAGNFRMPVLREYLMSGGICPVNRDTIDYLLSKNGSGNAIIIVVGGAAESLSSMPGKNAVTLKNR<br>KGFVKLALRHGADLVPTYSFGENEVYKQVIFEEGSWGRWVQKKFQKYIGFAPCIFHGR-<br>GLFSSDTWGLVPYSKPITTVVGEPITVPKLEHPTQKDIDLYHAMYMEALVKLFDNHKTKFGLPETEVLEVN
**>XP_008842455.1:1-388 diacylglycerol O-acyltransferase 2 [Nannospalax galili]**
MKTLIAAYSGVLRGERRAEAAHSENKKGGSSLSREGSGRWGTGSSIL-<br>SALQDIFSVTWLNRSKVEKQLQVISVLQWVLSFLVLGVACSVILMYTFCTDCWLIAVLYFTWLAFDWNTPKKGGRRS<br>QWVRNWAVWRYFRDYFPIQLVKTHNLLTTRNYIFGYHPHGIMGLGAFCNFSTEATEV-<br>SKKFPGIRPYLATLAGNFRIPVLREYLMSGGICPVNRDTIDYLLSKNGNGNAIIIVVGGAAESLSSMPGKNAVTLRNR<br>KGFVKLALRHGADLVPTYSFGENEVYKQVIFEEGSWGRWVQKKFQKYIGFAPCIFHGR-<br>GLFSSDTWGLVPYSKPITTVVGEPITIPKVEHPTQQDIDLYHAMYMEALVKLFDKHKTKFGLPETEVLEVN
**>XP_012600348.1:1-388 diacylglycerol O-acyltransferase 2 [Microcebus murinus]**
MKTLIAAYSGVLRGERRAEGARSASPNGGAALSREGSGRWGTGSSILSALQDLFSIT-<br>WLNRSKVEKQLQVISVLQWVLSFLVLGVACSVILMYTFCTDCWLIAVLYFTWLVFDWNTPKKGGRRSQWVRNWA<br>VWRYFRDYFPIQLVKTHNLLTTRNYIFGYHPHGIMGLGAFCNFSTEATEV-<br>SKKFPGIRPYLATLAGNFRMPVLREYLMSGGICPVNRDTIDYLLSKNGSGNAVIIVVGGAAESLSSMPGKNAVNLRN<br>RKGFVKLALRHGADLVPTYSFGENEVYKQVIFQEGSWGRWVQKKFQKYIGFAP-<br>CIFHGRGFFSSDTWGLVPYSKPITTVVGEPITIPKLEHPTQQDIDLYHAMYMEALVKLFDNHKTKFGLPETEVLEVN
**>XP_003780936.1:1-388 diacylglycerol O-acyltransferase 2 [Otolemur garnettii]**
MKTLIAAYSGVLRGERRAEAARSKSPKGGSALSREGSGRWGAGSSILSALQDLFSIT-<br>WLNRSKVEKQLQVISVLQWVLSFLVLGVACSAILMYTFCTDCWLIAVLYFTWLVFDWNTPKKGGRRSQWVRNWA<br>VWRYFRDYFPIQLVKTHNLLTTRNYILGYHPHGIMGLGAFCNFSTEATEV-<br>SKKFPGIRPYLATLAGNFRMPVLREYLMSGGICPVNRDTIDYLLSKNGTGNAIIIVVGGAAESLSSMPGKNAVTLRNR<br>KGFVKLALRHGADLVPTYSFGENEVYKQVIFQEGSWGRWVQKKFQKYIGFAPCIFHGR-<br>GLFSSDTWGLVPYSKPITTVVGEPITIPKLEYPTQQDIDLYHAMYMEALVKLFDSHKIKFGLPETEVLEVN
**>XP_021519710.1:1-388 diacylglycerol O-acyltransferase 2 isoform X1 [Meriones unguiculatus]**
MKTLIAAYSGVLRGERRNEAAGSENKNGGSALSRKGSGRWGTGSSIL-<br>SALQDIFSVTWLNRSKVEKQLQVISVLQWVLSFLVLGVACSVILMYTFCTDCWLIAVLYFTWLAFDWNTPKKGGRRS<br>QWVRNWAVWRYFRDYFPIQLVKTHNLLTTRNYIFGYHPHGIMGLGAFCNFSTEATEV-<br>SKKFPGIRPYLATLAGNFRMPVLREYLMSGGICPVNRDTIDYLLSKNGSGNAIIIVVGGAAESLSSMPGKNAVTLRNR<br>KGFVKLALRHGADLVPTYSFGENEVYKQVIFEEGSWGRWVQKKFQKYIGFAPCIFHGR-<br>GLFSSDTWGLVPYSKPITTVVGEPITIPKLEHPTQKDIDLYHAMYMEALVKLFDNHKTKFGLPETEVLEVN
**>XP_004683270.1:1-388 PREDICTED: diacylglycerol O-acyltransferase 2 [Condylura cristata]**
MKTLIAAYSGVLRGERRTPPDQSEGPKGGPVLSREGSRLWGTGSSILSALQDLFSIT-<br>WLNRSKVEKQLQVISVLQWVLSFLVLGVTCSVILMYIFCTDCWLIAVLYFTWLVFDWNTPKKGGRRSQWVRNWAV<br>WRYFRDYFPIQLVKTHNLLTTRNYIFGYHPHGIMGLGAFCNFSTEATEV-<br>SKKFPGIRPYLATLAGNFRMPVLREYLMSGGICPVNRDTIDYLLSKNGSGNAIIIVVGGAAESLSSMPGKNAVTLRNR<br>KGFVKLALRHGADLVPTYSFGENEVYKQVIFEEGSWGRWVQKKFQKYIGFAPCIFHGR-<br>GLFSSDTWGLVPYSKPITTVVGEPITVPKLEHPTQQDIDLYHAMYMEALVKLFDKHKTKFGLPETEVLEVN
**>XP_020011032.1:1-388 diacylglycerol O-acyltransferase 2 [Castor canadensis]**
MKTLIAAYSGVLRGERRAAATRSESSNGGSALSPQRSGRWGAGSSIL-<br>SALQDIFSVTWLNRSKVEKQLQVISVLQWVLSFLVLGVACSVILMYTFCTDCWLIAVLYFTWLAFDWNTPKKGGRRS<br>QWVRNWAVWRYFRDYFPIQLVKTHNLLTTRNYIFGYHPHGIMGLGAFCNFSTEATEV-<br>SKKFPGIRPYLATLAGNFRMPVLREYLMSGGICPVNRDTIDYLLSKNGSGNAIIIVVGGAAESLSSMPGKNAVTLRNR<br>KGFVKLALRHGADLVPTYSFGENEVYKQVIFEEGSWGRWVQKKFQKYIGFAPCIFHGR-<br>GLFSSDTWGLVPYSKPITTVVGEPITIPKLEHPTQQDIDLYHTMYVEALVKLFDKHKTKFGLLETEVLEVN
**>XP_017388511.1:1-382 diacylglycerol O-acyltransferase 2 [Cebus imitator]**

MKTLIAAYSGVLRGERRAKADPIQRSHGGPSLSREGSGRWGTGSSILSALQDLFSIT-<br>WINRSKVEKQLQVISVLQWVLSFLVMGVACSAILMYIFCTDCWLIAVLYFTWLVFDWNTPKKGGRRSQWVRNWAV<br>WRYFRDYFPIQLVKTHNLSTTRNYIFGYHPHGIMGLGAFCNFSTEATEV-<br>SKKFPGIRPYLATLAGNFRMPVLREYLMSGGICPVNRDTIDYLLSKNGSGNAIIIVVGGAAESLSSMPGKNAVTLRNR<br>KGFVKLALRHGADLVPVYSFGENEVYKQVIFEEGSWGRWVQKKFQKYIGFAP-<br>CIFHGQGLFSSNTWGLVPYSKPITTVVGEPITIPKLDIDLYHTMYMEALVKLFDKHKTKFGLPETEVLEVN
**>NP_001012345.1:1-388 diacylglycerol O-acyltransferase 2 [Rattus norvegicus]**
MKTLIAAYSGVLRGERRAEAARSENKNKGSALSREGSGRWGTGSSIL-<br>SALQDIFSVTWLNRSKVEKHLQVISVLQWVLSFLVLGVACSVILMYTFCTDCWLIAALYFTWLAFDWNTPKKGGRRS<br>QWVRNWAVWRYFRDYFPIQLVKTHNLLTTRNYIFGYHPHGIMGLGAFCNFSTEATEV-<br>SKKFPGIRPYLATLAGNFRMPVLREYLMSGGICPVNRDTIDYLLSKNGSGNAIVIVVGGAAESLSSMPGKNAVTLRNR<br>KGFVKLALRHGADLVPTYSFGENEVYKQVIFEEGSWGRWVQKKFQKYIGFAPCIFHGR-<br>GLFSSDTWGLVPYSKPITTVVGEPITVPKLEHPTQKDIDLYHTMYMEALVKLFDNHKTKFGLPETEVLEVN
**>XP_034343129.1:1-388 diacylglycerol O-acyltransferase 2 [Arvicanthis niloticus]**
MKTLIAAYSGVLRGERRAEAARNENKNKGSALSREGSGRWGTGSSIL-<br>SALQDIFSVTWLNRSKVEKQLQVISVLQWVLSFLVLGVACSVILMYTFCTDCWLIAVLYFTWLAFDWNTPKKGGRRS<br>QWVRNWAVWRYFRDYFPIQLVKTHNLLTTRNYIFGYHPHGIMGLGAFCNFSTEATEV-<br>SKKFPGIRPYLATLAGNFRMPVLREYLMSGGICPVNRDTIDYLLSKNGSGNAIIIVVGGAAESLSSMPGKNAVTLRSR<br>KGFVKLALRHGADLVPTYSFGENEVYKQVIFEEGSWGRWVQKKFQKYIGFAPCIFHGR-<br>GLFSSDTWGLVPYSKPITTVVGEPITVPKLEHPTQKDIDLYHSMYMEALVKLFDDHKTKFGLPETEVLEVN
**>XP_006141071.1:1-388 diacylglycerol O-acyltransferase 2 [Tupaia chinensis]**
MKTLIAAYSGVLRGERRAEAARSKNSNGGPALSREGSRRWGTGSSI-<br>LAALQDLFSVSWLNRSKVEKQLQVISVLQWVLSFLVLGVVCSIILMYTFCTDCWLIAVLYFTWLAFDWNTPKKGGRR<br>SQWVRNWAVWRYFRDYFPIQLVKTHNLLTTRNYIFGYHPHGIMGLGAFCNFSTEATEV-<br>SKKFPGIRPYLATLAGNFRFPVLREYLMSGGICPVNRDTIDYLLSKNGSGNAIIIVVGGAAESLSSMPGKNAVTLRNRK<br>GFVKLALRHGADLVPIYSFGENEVYQQVIFEEGSWGRWVQKKFQKYIGFAPCIFHGRGLF-<br>SADTWGLVPYSKPITTVVGEPITVPKLERPTQQDIDLYHAMYVEALVKLFDKHKTKFGLPETEVLEVN
**>XP_004632566.1:1-388 diacylglycerol O-acyltransferase 2 [Octodon degus]**
MKTLIAAYSGVLRGERRAKAARSESSSGRSVLTREGSGRWGTGSSIL-<br>SALQDLFSVTWLNRSKVEKQLQVISVLQWVLSFLVLGVACSAILMYTFCTDCWLIAVLYFTWLAFDWDTPKKGGRR<br>SQWVRNWAVWRYFRDYFPIQLVKTHNLPTTRNYIFGYHPHGIMGLGAFCNFSTEATEV-<br>SKKFPGIRPYLATLAGNFRMPVLREYLMSGGICPVNRDTIDYLLSKNGSGNAIIIVVGGAAESLSSMPGKNAVTLKNR<br>KGFVKLALRHGADLVPIYSFGENEVYKQVIFEEGSWGRWVQKKFQKYIGFAPCIFHGR-<br>GLFSSDTWGLVPYSKPITTVVGEPITIPKVEHPSQQDIDLYHAMYMDALVKLFDKHKTKFGLPETEVLEVN

Table S2. DGAT2 mutations reported on COSMIC separated by cancer type. For the coding region mutations, the amino acid substitution is shown (e.g. p.L59F) while for the non-coding ones the DNA coordinates are shown (e.g. c.430-889G>T). For some mutations, references are provided on COSMIC and they are indicated.

Cancer type	Mutation ID	References
Biliary Tract	p.L59F, p.D98G, p.S88N, c.430-889G>T, c.635-19C>T, c.809+27_809+28del,c.121+7383G>A, c.*565G>T	[1]
Bone	p.F370=, c.809+27_809+28del	[2,3]
Breast	p.E35K, p.E291K, p.I94V, p.A7G, c.121+82G>A, c.122-5056del, c.635-2A>T,c.121+3242A>G, c.122-1675C>G, c.122-3259C>G, c.*19T>C, c.809+27_809+28del,c.*11G>A, c.122-5278C>G, c.122-4081G>T, c.122-	[4]

	6138A>G, c.121+2300G>A, c.122-2287C>G, c.1013-736T>A, c.429+221G>A, c.122-880C>T, c.121+6815A>C,,c.429+2510G>C, c.429+2290G>C, c.429+2024G>A, c.122-7251T>A, c.122-5291T>C c.122-489G>A, c.121+5916C>G, c.635-246C>A, c.635-246C>A, c.251-990G>A c.429+1973G>C, c.429+1383G>T, c.429+1850G>A, c.429+742G>A,c.429+2108G>A, c.429+749G>C, c.429+2269G>C, c.429+1444G>A,c.429+1231G>A, c.429+932G>A, c.429+2434G>A, c.429+2421G>A,c.429+1965G>A	
Central Nervous System	p.G212C, p.A241V, p.P380L, p.A49T, p.A310T, c.1013-801C>T	[5,6]
Cervix	p.E35Q, p.G250=, p.D272H	N/A
Endometrium	p.R189W, p.G197D, p.Y358H, p.S175G, p.A7T, p.S294=, p.R154S, p.D52N, p.F377=,p.E177K, p.A310T, p.P215H, p.D371G, p.H163R, p.P329S, p.P380=, p.K227N,p.H163Pfs*81, c.*61A>C, c.*374C>A, c.*363G>A, c.*134T>C, c.*627A>G,c.*100G>T, c.*556A>T, c.*500C>T	N/A
Haematopoietic and Lymphoid	p.R297Q, p.A242Gfs*2, p.G164S, p.S230=, p.T375N, p.G43=, p.R134H,c.429+448G>A, c.250+2538T>Cc.429+1594C>T, c.122-6444C>G, c.*19T>C, c.1013-94G>A, c.1013-460A>G, c.*665C>T, c.*665C>T, c.*27A>G, c.121+2807T>A, c.251-851G>C, c.*19T>C	[7-10]
Kidney	p.D222V, p.P333=, p.G318R, c.251-1786C>T, c.121+6211C>T, c.121+1014C>T	[11,12]
Large Intestine	p.A241=, p.V132M, p.S79F, p.D323N, p.T57=, p.P380L, p.D356N, p.A242=,p.G378=, p.R268C, p.F290=, p.G296V, p.A7D, p.A241V, p.F370=, p.R13C, p.G231=,p.D356N, p.R259H, p.R134C, p.R317*, p.K146N, c.430-1395T>C, c.121+3748T>C,c.121+4931A>G	[13-18]
Liver	p.G293S, p.D21H, p.S244C, p.R134L, p.G326=, p.L267R, p.A242=, p.W126S,p.L78=, c.1012+49A>G, c.1012+736A>T, c.-184G>T, c.122-6772C>G,c.121+6015C>T,	[19]

	c.1012+49A>G, c.250+1796A>G, c.122-401C>T, c.358+46T>G,c.251-2116T>G, c.430-835C>A, c.251-1207C>T, c.*914G>T, c.430-2759C>A, c.-184G>T, c.-205G>A, c.121+131T>A, c.1012+613A>G, c.122-6772C>G, c.1013-91C>T,c.121+910T>A, c.1013-728A>T, c.122-1126G>A, c.-205G>A, c.121+1205G>T, c.-44G>T, c.430-930C>G, c.1013-834A>G, c.122-2961A>G, c.122-401C>T, c.430-37C>T, c.-205G>A, c.430-941T>A, c.122-3572A>G, c.122-6180T>G, c.122-3151G>A,c.429+1476C>A, c.635-69G>A, c.1013-496T>C, c.121+2255C>G, c.635-39A>T,c.251-418G>T, c.121+6874C>T, c.635-203G>C, c.429+1688A>G, c.121+718A>G,c.121+6923A>T, c.122-6433A>G, c.122-6824T>G, c.122-2919G>A, c.121+5114G>T,c.122-3740A>G, c.1013-496T>C, c.635-69G>A, c.122-4595del, c.430-2302A>G,c.1013-736T>A, c.1013-711T>G, c.-131C>T, c.121+6751C>T, c.122-6548G>C,c.358+46T>G, c.635-36G>T, c.122-3091C>G, c.121+477G>T, c.251-194C>A	
Lung	p.G270R, p.P141S, p.L245V, p.R297L, p.E291*, p.G270R, p.R205K, p.K301M,p.A241V, p.E283*, p.R257Q, p.W40*, p.E19D, p.L50H, p.A193S, p.G261C, c.*19T>C,c.*362C>T, c.430-139A>C, c.251-26C>G, c.250+180C>A, c.251-2349A>G	[20-22] [23-25]
Meninges	c.809+27_809+28del, c.809+27_809+28del	[26]
Oesophagus	p.A49V, p.A49=, p.V77=, p.Y139*, c.121+3498C>T, c.250+273T>A, c.121+5964C>T,c.122-2694G>A, c.1013-735T>A	[27-29]
Ovary	p.P215=, p.T108I, p.P215=, p.G36V, c.121+7311C>T, c.1012+932A>T,c.121+7311C>T, c.121+4392A>T, c.121+3396G>T, c.429+1452C>G, c.121+7311C>T, c.121+6451G>A, c.1013-795G>T, c.809+69C>T, c.430-621A>G, c.1012+931G>T, c.251-380G>C, c.121+5947C>G, c.430-1404C>G, c.121+1847C>A, c.121+6451G>A	N/A

Pancreas	p.F262V, c.*742G>C, c.1013-728A>T, c.*410C>G, c.430-325G>A, c.122-2486C>T, c.251-1636G>T, c.251-925C>T, c.122-2486C>T, c.122-4885C>T, c.121+251G>T, c.122-3052C>T, c.122-3372T>C, c.122-2486C>T, c.122-6243C>G, c.121+4577C>A, c.122-6035G>A, c.809+193G>A, c.122-3372T>C, c.250+1071G>T, c.121+3887C>G	N/A
Prostate	p.G167D, p.F377=, p.K264=, c.429+1944G>A, c.121+5990G>A, c.1012+205A>G, c.430-1458C>T, c.122-2752G>A, c.430-2138C>G	[30,31]
Skin	p.R137*, p.I355=, p.S294F, p.R200=, p.L81F, p.S331F, p.R137*, p.I289M, p.Q143*, p.L379=, p.F309=, p.I71F, p.R189Q, p.P215S, p.S321F, p.F80=, p.F80Y, p.R189L, p.I46=, p.I236=, p.P333L, p.N155S, p.R218W, p.F377=, p.P345H, p.R189=, p.F303=, p.W100L, p.S278=, p.I236=, p.I313=, p.F290=, p.G120D, p.R39K, p.P345H, c.250+1915G>A, c.359-8C>T, c.358+1G>A, c.251-36C>T	[32-40]
Soft Tissue	p.D21=, p.S278F	N/A
Stomach	p.W126R, p.R134C, p.F136V, p.S44Y, p.F377=, p.N228Y, p.R297*, c.635-41C>A, c.359-169C>T, c.-38C>T, c.809+27_809+28del, c.*16C>A, c.809+27_809+28del, c.-23C>T, c.809+27_809+28del, c.809+27_809+28del, c.121+69G>A	[41-43]
Thyroid	p.D21E, p.S88N, p.S88N, p.G41S, p.G41S, p.D21E	N/A
Upper Aerodigestive Tract	p.P380L, p.F290=, p.F314S, p.Q51K, p.S322C, p.R200=, p.R218Q, p.E243Q, p.E340K, c.121+1779G>T	[44-46]
Urinary Tract	p.P275=, p.E15*, p.L319=	N/A
Not Specified	p.P141S, p.G280Efs*9, p.P141S	[47]

Table S3–S4 are found separately attached as Excel Files.

References

1. Kim, Y.-H.; Hong, E.-K.; Kong, S.-Y.; Han, S.-S.; Kim, S.-H.; Rhee, J.-K.; Hwang, S.-K.; Park, S.-J.; Kim, T.-M. Two classes of intrahepatic cholangiocarcinoma defined by relative abundance of mutations and copy number alterations. *Oncotarget* **2016**, *7*, 23825–23836, doi:10.18632/oncotarget.8183.
2. Perry, J.A.; Kiezun, A.; Tonzi, P.; Van Allen, E.M.; Carter, S.L.; Baca, S.C.; Cowley, G.S.; Bhatt, A.S.; Rheinbay, E.; Pedamallu, C.S.; et al. Complementary genomic approaches highlight the PI3K/mTOR pathway as a common vulnerability in osteosarcoma. *Proc. Natl. Acad. Sci.* **2014**, *111*, E5564–E5573, doi:10.1073/pnas.1419260111.
3. Crompton, B.D.; Stewart, C.; Taylor-Weiner, A.; Alexe, G.; Kurek, K.C.; Calicchio, M.L.; Kiezun, A.; Carter, S.L.; Shukla, S.A.; Mehta, S.S.; et al. The Genomic Landscape of Pediatric Ewing Sarcoma. *Cancer Discov.* **2014**, *4*, 1326–1341, doi:10.1158/2159-8290.cd-13-1037.
4. Lefebvre, C.; Bachelot, T.; Filleron, T.; Pedrero, M.; Campone, M.; Soria, J.-C.; Massard, C.; Lévy, C.; Arnedos, M.; Lacroix-Triki, M.; et al. Mutational Profile of Metastatic Breast Cancers: A Retrospective Analysis. *PLoS Med.* **2016**, *13*, e1002201, doi:10.1371/journal.pmed.1002201.

5. Lee, J.-K.; Wang, J.; Sa, J.K.; Ladewig, E.; Lee, H.-O.; Lee, I.-H.; Kang, H.J.; Rosenbloom, D.S.; Camara, P.G.; Liu, Z.; et al. Spatiotemporal genomic architecture informs precision oncology in glioblastoma. *Nat. Genet.* **2017**, *49*, 594–599, doi:10.1038/ng.3806.
6. Aihara, K.; Mukasa, A.; Nagae, G.; Nomura, M.; Yamamoto, S.; Ueda, H.; Tatsuno, K.; Shibahara, J.; Takahashi, M.; Momose, T.; et al. Genetic and epigenetic stability of oligodendrogliomas at recurrence. *Acta Neuropathol. Commun.* **2017**, *5*, 1–11, doi:10.1186/s40478-017-0422-z.
7. Walter, M.J.; Shen, D.; Ding, L.; Shao, J.; Koboldt, D.C.; Chen, K.; Larson, D.; McLellan, M.D.; Dooling, D.; Abbott, R.; et al. Clonal Architecture of Secondary Acute Myeloid Leukemia. *New Engl. J. Med.* **2012**, *366*, 1090–1098, doi:10.1056/nejmoa1106968.
8. Tessoulin, B.; Moreau-Aubry, A.; Descamps, G.; Bougie, P.G.; Maïga, S.; Gaignard, A.; Chiron, D.; Ménoret, E.; le Gouill, S.; Moreau, P.; et al. Whole-exon sequencing of human myeloma cell lines shows mutations related to myeloma patients at relapse with major hits in the DNA regulation and repair pathways. *J. Hematol. Oncol.* **2018**, *11*, 1–13, doi:10.1186/s13045-018-0679-0.
9. Zhang, J.; McCastlain, K.; Yoshihara, H.; Xu, B.; Chang, Y.; Churchman, M.L.; Wu, G.; Li, Y.; Wei, L.; Iacobucci, I.; et al. Deregulation of DUX4 and ERG in acute lymphoblastic leukemia. *Nat. Genet.* **2016**, *48*, 1481–1489, doi:10.1038/ng.3691.
10. Holmfeldt, L.; Wei, L.; Diaz-Flores, E.; Walsh, M.; Zhang, J.; Ding, L.; Payne-Turner, D.; Churchman, M.; Hagström-Andersson, A.; Chen, S.-C.; et al. The genomic landscape of hypodiploid acute lymphoblastic leukemia. *Nat. Genet.* **2013**, *45*, 242–252, doi:10.1038/ng.2532.
11. Gerlinger, M.; Horswell, S.; Larkin, J.; Rowan, A.J.; Salm, M.; Varela, I.; Fisher, R.; McGranahan, N.; Matthews, N.; Santos, C.R.; et al. Genomic architecture and evolution of clear cell renal cell carcinomas defined by multiregion sequencing. *Nat. Genet.* **2014**, *46*, 225–233, doi:10.1038/ng.2891.
12. Sato, Y.; Yoshizato, T.; Shiraishi, Y.; Maekawa, S.; Okuno, Y.; Kamura, T.; Shimamura, T.; Sato-Otsubo, A.; Nagae, G.; Suzuki, H.; et al. Integrated molecular analysis of clear-cell renal cell carcinoma. *Nat. Genet.* **2013**, *45*, 860–867, doi:10.1038/ng.2699.
13. Giannakis, M.; Mu, X.J.; Shukla, S.A.; Qian, Z.R.; Cohen, O.; Nishihara, R.; Bahl, S.; Cao, Y.; Amin-Mansour, A.; Yamauchi, M.; et al. Genomic Correlates of Immune-Cell Infiltrates in Colorectal Carcinoma. *Cell Rep.* **2016**, *15*, 857–865, doi:10.1016/j.celrep.2016.03.075.
14. Mouradov, D.; Sloggett, C.; Jorissen, R.; Love, C.G.; Li, S.; Burgess, A.W.; Arango, D.; Strausberg, R.L.; Buchanan, D.; Wormald, S.; et al. Colorectal Cancer Cell Lines Are Representative Models of the Main Molecular Subtypes of Primary Cancer. *Cancer Res.* **2014**, *74*, 3238–3247, doi:10.1158/0008-5472.can-14-0013.
15. Seshagiri, S.; Stawiski, E.W.; Durinck, S.; Modrusan, Z.; Storm, E.E.; Conboy, C.B.; Chaudhuri, S.; Guan, Y.; Janakiraman, V.; Jaiswal, B.S.; et al. Recurrent R-spondin fusions in colon cancer. *Nature* **2012**, *488*, 660–664, doi:10.1038/nature11282.
16. Giannakis, M.; Hodis, E.; Mu, X.J.; Yamauchi, M.; Rosenbluh, J.; Cibulskis, K.; Saksena, G.; Lawrence, M.S.; Qian, Z.R.; Nishihara, R.; et al. RNF43 is frequently mutated in colorectal and endometrial cancers. *Nat. Genet.* **2014**, *46*, 1264–1266, doi:10.1038/ng.3127.
17. Liu, Z.; Yang, C.; Li, X.; Luo, W.; Roy, B.; Xiong, T.; Zhang, X.; Yang, H.; Wang, J.; Ye, Z.; et al. The landscape of somatic mutation in sporadic Chinese colorectal cancer. *Oncotarget* **2018**, *9*, 27412–27422, doi:10.18632/oncotarget.25287.
18. Abaan, O.D.; Polley, E.C.; Davis, S.; Zhu, Y.J.; Bilke, S.; Walker, R.L.; Pineda, M.; Gindin, Y.; Jiang, Y.; Reinhold, W.; et al. The Exomes of the NCI-60 Panel: A Genomic Resource for Cancer Biology and Systems Pharmacology. *Cancer Res.* **2013**, *73*, 4372–4382, doi:10.1158/0008-5472.can-12-3342.
19. Wang, A.; Wu, L.; Lin, J.; Han, L.; Bian, J.; Wu, Y.; Robson, S.C.; Xue, L.; Ge, Y.; Sang, X.; et al. Whole-exome sequencing reveals the origin and evolution of hepato-cholangiocarcinoma. *Nat. Commun.* **2018**, *9*, 894, doi:10.1038/s41467-018-03276-y.
20. Liu, J.; Lee, W.; Jiang, Z.; Chen, Z.; Jhunjhunwala, S.; Haverty, P.M.; Gnad, F.; Guan, Y.; Gilbert, H.N.; Stinson, J.; et al. Genome and transcriptome sequencing of lung cancers reveal diverse mutational and splicing events. *Genome Res.* **2012**, *22*, 2315–2327, doi:10.1101/gr.140988.112.
21. Li, C.; Gao, Z.; Li, F.; Li, X.; Sun, Y.; Wang, M.; Li, D.; Wang, R.; Li, F.; Fang, R.; et al. Whole Exome Sequencing Identifies Frequent Somatic Mutations in Cell-Cell Adhesion Genes in Chinese Patients with Lung Squamous Cell Carcinoma. *Sci. Rep.* **2015**, *5*, 14237, doi:10.1038/srep14237.
22. Rudin, C.M.; Durinck, S.; Stawiski, E.W.; Poirier, J.; Modrusan, Z.; Shames, D.S.; Bergbower, E.; Guan, Y.; Shin, J.; Guillory, J.; et al. Comprehensive genomic analysis identifies SOX2 as a frequently amplified gene in small-cell lung cancer. *Nat. Genet.* **2012**, *44*, 1111–1116, doi:10.1038/ng.2405.
23. McMillan, E.A.; Ryu, M.-J.; Diep, C.H.; Mendiratta, S.; Clemenceau, J.; Vaden, R.M.; Kim, J.-H.; Motoyaji, T.; Covington, K.R.; Peyton, M.; et al. Chemistry-First Approach for Nomination of Personalized Treatment in Lung Cancer. *Cell* **2018**, *173*, 864–878.e29, doi:10.1016/j.cell.2018.03.028.
24. Imielinski, M.; Berger, A.H.; Hammerman, P.S.; Hernandez, B.; Pugh, T.J.; Hodis, E.; Cho, J.; Suh, J.; Capelletti, M.; Sivachenko, A.; et al. Mapping the Hallmarks of Lung Adenocarcinoma with Massively Parallel Sequencing. *Cell* **2012**, *150*, 1107–1120, doi:10.1016/j.cell.2012.08.029.

25. Biswas, R.; Gao, S.; Cultraro, C.M.; Maity, T.K.; Venugopalan, A.; Abdullaev, Z.; Shaytan, A.; Carter, C.A.; Thomas, A.; Rajan, A.; et al. Genomic profiling of multiple sequentially acquired tumor metastatic sites from an "exceptional responder" lung adenocarcinoma patient reveals extensive genomic heterogeneity and novel somatic variants driving treatment response. *Mol. Case Stud.* **2016**, *2*, a001263, doi:10.1101/mcs.a001263.
26. Bi, W.L.; Greenwald, N.F.; Abedalthagafi, M.; Wala, J.; Gibson, W.J.; Agarwalla, P.K.; Horowitz, P.; Schumacher, S.E.; Esaulova, E.; Mei, Y.; et al. Genomic landscape of high-grade meningiomas. *npj Genom. Med.* **2017**, *2*, 1–14, doi:10.1038/s41525-017-0014-7.
27. Gao, Y.-B.; Chen, Z.-L.; Li, J.-G.; Hu, X.-D.; Shi, X.-J.; Sun, Z.-M.; Zhang, F.; Zhao, Z.-R.; Li, Z.-T.; Liu, Z.-Y.; et al. Genetic landscape of esophageal squamous cell carcinoma. *Nat. Genet.* **2014**, *46*, 1097–1102, doi:10.1038/ng.3076.
28. Chang, J.; Tan, W.; Ling, Z.; Xi, R.; Shao, M.; Chen, M.; Luo, Y.; Zhao, Y.; Liu, Y.; Huang, X.; et al. Genomic analysis of oesophageal squamous-cell carcinoma identifies alcohol drinking-related mutation signature and genomic alterations. *Nat. Commun.* **2017**, *8*, 15290, doi:10.1038/ncomms15290.
29. Dulak, A.M.; Stojanov, P.; Peng, S.; Lawrence, M.S.; Fox, C.; Stewart, C.; Bandla, S.; Imamura, Y.; E Schumacher, S.; Shefler, E.; et al. Exome and whole-genome sequencing of esophageal adenocarcinoma identifies recurrent driver events and mutational complexity. *Nat. Genet.* **2013**, *45*, 478–486, doi:10.1038/ng.2591.
30. Petrovics, G.; Li, H.; Stümpel, T.; Tan, S.-H.; Young, D.; Katta, S.; Li, Q.; Ying, K.; Klocke, B.; Ravindranath, L.; et al. A novel genomic alteration of LSAMP associates with aggressive prostate cancer in African American men. *EBioMedicine* **2015**, *2*, 1957–1964, doi:10.1016/j.ebiom.2015.10.028.
31. Barbieri, C.; Baca, S.C.; Lawrence, M.S.; Demichelis, F.; Blattner, M.; Theurillat, J.-P.; A White, T.; Stojanov, P.; Van Allen, E.; Stransky, N.; et al. Exome sequencing identifies recurrent SPOP, FOXA1 and MED12 mutations in prostate cancer. *Nat. Genet.* **2012**, *44*, 685–689, doi:10.1038/ng.2279.
32. Berger, M.F.; Hodis, E.; Heffernan, T.P.; Deribe, Y.L.; Lawrence, M.S.; Protopopov, A.; Ivanova, E.; Watson, I.; Nickerson, E.; Ghosh, P.; et al. Melanoma genome sequencing reveals frequent PREX2 mutations. *Nat. Cell Biol.* **2012**, *485*, 502–506, doi:10.1038/nature11071.
33. Sharpe, H.; Pau, G.; Dijkgraaf, G.J.; Basset-Seguin, N.; Modrusan, Z.; Januario, T.; Tsui, V.; Durham, A.B.; Dlugosz, A.A.; Haverty, P.M.; et al. Genomic Analysis of Smoothened Inhibitor Resistance in Basal Cell Carcinoma. *Cancer Cell* **2015**, *27*, 327–341, doi:10.1016/j.ccell.2015.02.001.
34. Shain, A.H.; Garrido, M.; Botton, T.; Talevich, E.; Yeh, I.; Sanborn, J.Z.; Chung, J.; Wang, N.J.; Kakavand, H.; Mann, G.; et al. Exome sequencing of desmoplastic melanoma identifies recurrent NFKBIE promoter mutations and diverse activating mutations in the MAPK pathway. *Nat. Genet.* **2015**, *47*, 1194–1199, doi:10.1038/ng.3382.
35. Pickering, C.R.; Zhou, J.H.; Lee, J.J.; Drummond, J.A.; Peng, S.A.; Saade, R.E.; Tsai, K.Y.; Curry, J.L.; Tetzlaff, M.T.; Lai, S.Y.; et al. Mutational Landscape of Aggressive Cutaneous Squamous Cell Carcinoma. *Clin. Cancer Res.* **2014**, *20*, 6582–6592, doi:10.1158/1078-0432.ccr-14-1768.
36. Krauthammer, M.; Kong, Y.; Bacchiocchi, A.; Evans, P.; Pornputtapong, N.; Wu, C.; McCusker, J.; Ma, S.; Cheng, E.; Straub, R.; et al. Exome sequencing identifies recurrent mutations in NF1 and RASopathy genes in sun-exposed melanomas. *Nat. Genet.* **2015**, *47*, 996–1002, doi:10.1038/ng.3361.
37. Hayward, N.K.; Wilmott, J.S.; Waddell, N.; Johansson, P.A.; Field, M.A.; Nones, K.; Patch, A.-M.; Kakavand, H.; Alexandrov, L.B.; Burke, H.; et al. Whole-genome landscapes of major melanoma subtypes. *Nature* **2017**, *545*, 175–180, doi:10.1038/nature22071.
38. Heidenreich, B.; Denisova, E.; Rachakonda, S.; Sanmartín, O.; Dereani, T.; Hosen, I.; Nagore, E.; Kumar, R. Genetic alterations in seborrheic keratoses. *Oncotarget* **2017**, *8*, 36639–36649, doi:10.18632/oncotarget.16698.
39. South, A.P.; Purdie, K.J.; Watt, S.A.; Haldenby, S.; Breems, N.Y.D.; Dimon, M.; Arron, S.; Kluk, M.J.; Aster, J.C.; McHugh, A.; et al. NOTCH1 Mutations Occur Early during Cutaneous Squamous Cell Carcinogenesis. *J. Investig. Dermatol.* **2014**, *134*, 2630–2638, doi:10.1038/jid.2014.154.
40. González-Vela, M.D.C.; Curiel-Olmo, S.; Derdak, S.; Beltran, S.; Santibáñez, M.; Martinez, N.; Castillo-Trujillo, A.; Gut, M.; Sánchez-Pacheco, R.; Almaraz, C.; et al. Shared Oncogenic Pathways Implicated in Both Virus-Positive and UV-Induced Merkel Cell Carcinomas. *J. Investig. Dermatol.* **2017**, *137*, 197–206, doi:10.1016/j.jid.2016.08.015.
41. Wang, K.; Yuen, S.T.; Xu, J.; Lee, S.P.; Yan, H.N.H.; Shi, S.T.; Siu, H.C.; Deng, S.; Chu, K.M.; Law, S.; et al. Whole-genome sequencing and comprehensive molecular profiling identify new driver mutations in gastric cancer. *Nat. Genet.* **2014**, *46*, 573–582, doi:10.1038/ng.2983.
42. Liu, J.; McCleland, M.; Stawiski, E.W.; Gnad, F.; Mayba, O.; Haverty, P.M.; Durinck, S.; Chen, Y.-J.; Klijn, C.; Jhunjhunwala, S.; et al. Integrated exome and transcriptome sequencing reveals ZAK isoform usage in gastric cancer. *Nat. Commun.* **2014**, *5*, 3830, doi:10.1038/ncomms4830.
43. Kim, T.-M.; Jung, S.-H.; Kim, M.S.; Baek, I.-P.; Park, S.-W.; Lee, S.H.; Lee, H.H.; Kim, S.S.; Chung, Y.-J.; Lee, S.H. The mutational burdens and evolutionary ages of early gastric cancers are comparable to those of advanced gastric cancers. *J. Pathol.* **2014**, *234*, 365–374, doi:10.1002/path.4401.
44. Fadlullah, M.Z.H.; Chiang, I.K.-N.; Dionne, K.R.; Yee, P.S.; Gan, C.P.; Sam, K.K.; Tiong, K.H.; Ng, K.-W.; Martin, D.; Lim, K.P.; et al. Genetically-defined novel oral squamous cell carcinoma cell lines for the development of molecular therapies. *Oncotarget* **2016**, *7*, 27802–27818, doi:10.18632/oncotarget.8533.

45. Li, Y.Y.; Chung, G.T.Y.; Lui, V.W.Y.; To, K.-F.; Ma, B.B.Y.; Chow, C.; K, S.W.J.; Yip, K.; Seo, J.; Hui, E.P.; et al. Exome and genome sequencing of nasopharynx cancer identifies NF-κB pathway activating mutations. *Nat. Commun.* **2017**, *8*, 1–10, doi:10.1038/ncomms14121.
46. Pickering, C.; Zhang, J.; Yoo, S.Y.; Bengtsson, L.; Moorthy, S.; Neskey, D.M.; Zhao, M.; Alves, M.V.O.; Chang, K.; Drummond, J.; et al. Integrative Genomic Characterization of Oral Squamous Cell Carcinoma Identifies Frequent Somatic Drivers. *Cancer Discov.* **2013**, *3*, 770–781, doi:10.1158/2159-8290.cd-12-0537.
47. Van Allen, E.; Wagle, N.; Sucker, A.; Treacy, D.J.; Johannessen, C.M.; Goetz, E.M.; Place, C.S.; Taylor-Weiner, A.; Whittaker, S.; Kryukov, G.; et al. The Genetic Landscape of Clinical Resistance to RAF Inhibition in Metastatic Melanoma. *Cancer Discov.* **2014**, *4*, 94–109, doi:10.1158/2159-8290.cd-13-0617.
